# Native Size-Exclusion Chromatography–Based Mass Spectrometry Reveals New Components of the Early Heat Shock Protein 90 Inhibition Response Among Limited Global Changes

**DOI:** 10.1016/j.mcpro.2022.100485

**Published:** 2022-12-20

**Authors:** Rahul S. Samant, Silvia Batista, Mark Larance, Bugra Ozer, Christopher I. Milton, Isabell Bludau, Estelle Wu, Laura Biggins, Simon Andrews, Alexia Hervieu, Harvey E. Johnston, Bissan Al-Lazikhani, Angus I. Lamond, Paul A. Clarke, Paul Workman

**Affiliations:** 1 Centre for Cancer Drug Discovery, The Institute of Cancer Research, London, United Kingdom; 2 Signalling Programme, The Babraham Institute, Cambridge, United Kingdom; 3 Centre for Gene Regulation & Expression, University of Dundee, Dundee, United Kingdom; 4 Department of Proteomics and Signal Transduction, Max Planck Institute of Biochemistry, Martinsried, Germany; 5 Bioinformatics Group, The Babraham Institute, Cambridge, United Kingdom; 6 Department of Genomic Medicine, The University of Texas MD Anderson Cancer Center, Houston, Texas, USA

**Keywords:** molecular chaperone, heat shock protein, protein complexes, proteomics, HSP90 inhibitor, tanespimycin, BSA, bovine serum albumin, CDK, cyclin-dependent kinase, CORUM, comprehensive resource of mammalian protein complexes, DDA, Data-dependent acquisition, DEP, differential enrichment analysis of proteomics data, DIA, data-independent acquisition, DP, differential protein, FDR, false discovery rate, GO, gene ontology, HSP90, heat shock protein 90, HSP90i, HSP90 inhibitor, IDH, isocitrate dehydrogenase, LFQ, label-free quantitation, LUMIER, luminescence-based mammalian interactome mapping, MS, mass spectrometry, NTC, nontargeting control, SEC, size-exclusion chromatography, SEC-IB, size-exclusion chromatography–coupled immunoblotting, SEC-MS, size-exclusion chromatography–coupled mass spectrometry, SILAC, stable isotope labeling with amino acids in cell culture, STIP1/HOP, stress-induced phosphoprotein 1/ Hsc70/Hsp90-organizing protein, STRING, Search Tool for the Retrieval of Interacting Genes, TBS-T, Tris-buffered saline with 1% Tween-20, TRiC, Tailless complex polypeptide 1 ring complex

## Abstract

The molecular chaperone heat shock protein 90 (HSP90) works in concert with co-chaperones to stabilize its client proteins, which include multiple drivers of oncogenesis and malignant progression. Pharmacologic inhibitors of HSP90 have been observed to exert a wide range of effects on the proteome, including depletion of client proteins, induction of heat shock proteins, dissociation of co-chaperones from HSP90, disruption of client protein signaling networks, and recruitment of the protein ubiquitylation and degradation machinery—suggesting widespread remodeling of cellular protein complexes. However, proteomics studies to date have focused on inhibitor-induced changes in total protein levels, often overlooking protein complex alterations. Here, we use size-exclusion chromatography in combination with mass spectrometry (SEC-MS) to characterize the early changes in native protein complexes following treatment with the HSP90 inhibitor tanespimycin (17-AAG) for 8 h in the HT29 colon adenocarcinoma cell line. After confirming the signature cellular response to HSP90 inhibition (*e.g.*, induction of heat shock proteins, decreased total levels of client proteins), we were surprised to find only modest perturbations to the global distribution of protein elution profiles in inhibitor-treated HT29 cells at this relatively early time-point. Similarly, co-chaperones that co-eluted with HSP90 displayed no clear difference between control and treated conditions. However, two distinct analysis strategies identified multiple inhibitor-induced changes, including known and unknown components of the HSP90-dependent proteome. We validate two of these—the actin-binding protein Anillin and the mitochondrial isocitrate dehydrogenase 3 complex—as novel HSP90 inhibitor-modulated proteins. We present this dataset as a resource for the HSP90, proteostasis, and cancer communities (https://www.bioinformatics.babraham.ac.uk/shiny/HSP90/SEC-MS/), laying the groundwork for future mechanistic and therapeutic studies related to HSP90 pharmacology. Data are available *via* ProteomeXchange with identifier PXD033459.

## Highlights

• Native SEC-MS of HT29 colon cancer cells treated with HSP90 inhibitor tanespimycin.
• Limited global changes to protein SEC fractionation profiles upon HSP90 inhibition.
• Novel HSP90-dependent proteins include Anillin and mitochondrial IDH3 complex.
• SEC-MS profiles of 4645 proteins ± HSP90 inhibition available to explore online.

## Graphical Abstract

**Figure:**
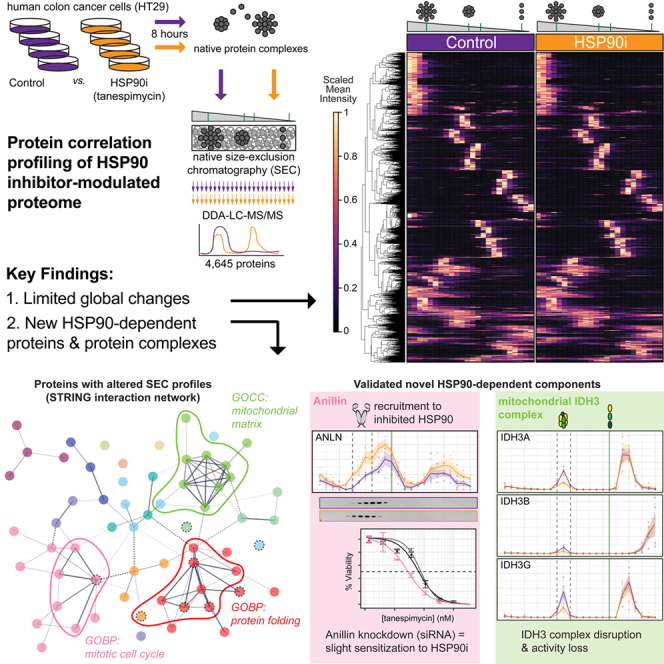

## In Brief

Samant & Batista *et al*. use native SEC-MS to characterize how protein complexes are modulated in human cancer cells treated with tanespimycin, a clinically developed inhibitor of the molecular chaperone HSP90. Among 4645 proteins identified across at least three of the four replicates, the authors unexpectedly find only limited changes to global protein distributions, rather than proteome-wide remodeling, following HSP90 inhibition. Nevertheless, several novel HSP90-dependent components are identified through this dataset, including Anillin and mitochondrial IDH3.

The molecular chaperone Heat Shock Protein 90 (HSP90) is required for the stabilization and activation of around 300 client proteins (see http://www.picard.ch/downloads/Hsp90interactors.pdf for the latest client list), many of which are oncogenic kinases that are mutated and/or hyper-activated in cancer (1). Furthermore, HSP90 may act as an ‘enabler’ of oncogenesis and malignant progression, potentially supporting tumor heterogeneity and contributing to drug resistance (2). Pharmacologic inhibitors of HSP90 have therefore been pursued as anticancer agents, either alone or in combination with other drugs. Despite mixed efficacy and tolerance in clinical trials (3, 4), there is continuing interest in HSP90 as a pharmacologic cancer target (5, 6), highlighted by the approval in June 2022 of one HSP90 inhibitor for chemotherapy-relapsed gastro-intestinal stromal tumors in Japan (7, 8). HSP90 inhibition has also shown potential beyond the cancer field, for example, as a broad-spectrum antiviral approach (9) (including activity against SARS-CoV2 (10)), and as a gero-protection strategy for healthier aging (11, 12). Therefore, improving our understanding of the molecular responses to HSP90 inhibition at a global, proteome-wide scale could inform rational strategies for patient selection and stratification across a variety of pathologies.

Global approaches could also help clarify the mechanisms underlying the tumor selectivity of HSP90 inhibitors—a phenomenon that has long been a matter of debate (13, 14). Given the number of oncoproteins that make up the HSP90 client list, a major rationale for deploying HSP90 inhibitors in cancer is the destabilization and disruption of signaling networks critical for oncogenesis and malignant progression. However, several alternative or additional mechanisms have been proposed, including the tumor-selective accumulation of several different HSP90 inhibitors as well as their much higher affinity for the hyper-activated HSP90 complexes specifically observed in tumor cells (13, 15, 16). This latter stress-associated assembly of high molecular-weight complexes, containing multiple chaperones and co-chaperones—more recently referred to as the ‘epichaperome’—may potentially be more predictive of patient response than expression levels of the chaperones or their individual oncogenic protein clients *per se* (16). Given the clinical interest in exploiting disease-altered states of epichaperome and proteostasis networks (17), unbiased system-wide analysis of how such higher-order protein assemblies are perturbed by HSP90 inhibitors and other proteostasis-modulating agents could be valuable for maximizing therapeutic benefit.

To date, proteomic studies aiming to characterize the HSP90-dependent proteome can be separated broadly into two categories (18). The first set of these comprise mass spectrometry (MS)–based comparative proteomics to identify proteins whose abundances change following HSP90 inhibitor treatment (19, 20, 21, 22, 23). While this approach undoubtedly has been fruitful, it misses client proteins whose levels do not change drastically, have slower degradation kinetics, or form nonfunctional oligomers/aggregates. It also ignores functionally consequential alterations in protein complexes whose total abundances would not be expected to change, including components of epichaperome assemblies (*e.g.*, co-chaperones, ubiquitin-modifying enzymes), as well as a diverse range of protein complexes reliant on HSP90 for their correct assembly and maintenance (24, 25).

Some of the limitations inherent in abundance-based comparative proteomics can be addressed through the second category of proteomics approaches, which employ direct ‘interactomics’ combining affinity-based assays with MS (26) or high-content fluorimetry (27) as a readout (18). However, these bait:prey-based techniques are also limited in scope. They are mostly unsuitable for detecting weak or highly labile interactions, and alterations observed in a protein:protein interaction following inhibitor treatment could be confounded by inhibitor-induced changes in the protein’s total abundance—thus making it difficult to interpret the data without extensive validation. Furthermore, indirect or downstream effects on protein complexes that do not interact with the HSP90 machinery are ignored.

Ideally, studies would incorporate the strengths of both approaches, allowing global identification of proteins that change in absolute abundance and/or in their distribution across different protein complexes—all in a single experiment. One potential solution is native size-exclusion chromatography–coupled mass spectrometry (SEC-MS), first employing size-exclusion chromatography (SEC) to separate protein complexes from a cell homogenate into different fractions according to their molecular weight, followed by bottom-up MS of each individual fraction (28, 29, 30). Importantly, SEC-MS allows analysis of endogenous protein complexes in cells without having to rely on affinity-tagged bait and/or overexpression systems, which have the potential to introduce artefacts. In this way, SEC-MS provides native molecular weight–based elution profiles, together with total abundance, at a proteome-wide level.

Here, we performed SEC-MS to characterize global changes to native protein complex distributions upon HSP90 inhibition with the geldanamycin-derivative tanespimycin (17-AAG) in the HT29 human colon adenocarcinoma cell line. We chose a tanespimycin concentration (62.5 nM) demonstrated to trigger the molecular signature of HSP90 inhibition (*e.g.*, HSP70 induction) in this cell line, but at an early enough treatment time (8 h) that the majority of client degradation had yet to take place—thereby allowing us to detect remodeling of protein complexes that depend on HSP90 for their assembly and/or maintenance (31). We identified 6427 unique proteins overall, including 4645 proteins in at least three of the four biological replicates. Known members of well-characterized protein complexes displayed similar SEC-MS elution profiles. We were surprised to find minimal changes to the profiles of most identified proteins following HSP90 inhibition—including co-chaperones that dissociated from HSP90 clients under the same treatment conditions as used in previous immunoprecipitation studies. The lack of changes to co-chaperones detected by SEC-MS—which was confirmed by independent SEC-Immunoblotting (SEC-IB)—was not due to a lack of target engagement, as the molecular signature of HSP90 inhibition was observed throughout our experiments. Nevertheless, we used two distinct analysis strategies to identify proteins and protein complexes whose SEC-MS profiles changed robustly in our dataset. These included several proteins previously characterized as being HSP90-dependent, as well as numerous novel hits—two of which we validated for biological importance. We present this dataset as a resource to the HSP90, proteostasis, and cancer communities (available to explore as a web-based Shiny app at https://www.bioinformatics.babraham.ac.uk/shiny/HSP90/SEC-MS/), providing novel candidates for further mechanistic and therapeutic studies.

## Experimental Procedures

### Cell Culture

HT29 human colon adenocarcinoma, HCT116 human colon carcinoma, and BT474 human breast ductal carcinoma cells purchased from ATCC (LGC Promochem) were cultured in Dulbecco’s modified Eagle’s Medium (Invitrogen) and supplemented with 10% fetal calf serum (PAA Laboratories), 2 mM L-glutamine, 0.1 mM non-essential amino acids, and 100 U of penicillin and streptomycin (all from Invitrogen) at 37 °C in a humidified incubator with 5% CO_2_ and subcultured at 70% confluency. Cells were confirmed as mycoplasma-free using the Venor *Mycoplasma* PCR Detection Kit (Minerva Biolabs) and were authenticated by short tandem repeat DNA profiling.

### Experimental Design and Statistical Rationale

Four biological replicates were prepared for SEC-MS analysis, based on the variance detected in our previous experiments using SEC-MS (29, 32). We had previously shown that the concentration of tanespimycin used here was sufficient to detect the signature response of HSP90 inhibition in the same cell line (31). For each biological replicate, tanespimycin treatment was always performed in parallel with dimethyl sulfoxide (DMSO) vehicle–treated control (*i.e.*, ‘Control-1’ and ‘HSP90 inhibitor (HSP90i)-1’ were treated in parallel, ‘Control-2’ with ‘HSP90i-2’, etc.). To create an elution profile for an individual protein in each of the four biological replicates in each experimental condition (Control or HSP90i), we used the MaxQuant label-free quantitation (LFQ) algorithm (33).

### Compound Treatment and Cell Lysis for SEC

Five 15 cm dishes (50% confluent) of HT29 cells were treated with 62.5 nM tanespimycin (Invivogen) (equivalent to 5× GI_50_ for the cell line) or mock-treated with equivalent volume of DMSO. The GI_50_ concentration for the cell line was determined by 96 h sulforhodamine B assay (34) and defined as the drug concentration that reduced the mean absorbance at 540 nm to 50% of vehicle-treated controls. After 8 h, the cells were scraped on ice in 500 μl of ice-cold PBS containing cOmplete Protease Inhibitor Cocktail EDTA-free (Roche) and PhosStop (Roche). The collected cells were sonicated with a Diagenode Bioruptor (30 cycles: 30 s on, 30 s off) at 4 °C and then centrifuged at 17,000*g* for 10 min at 4 °C. Samples were filtered through 0.45 μm Ultrafree-MC centrifugal filter units (Millipore) at 12,000*g* for 10 min.

### SEC, Enzymatic Digestion, and Peptide Clean-Up

Using a Dionex UltiMate 3000 HPLC system (Thermo Fisher Scientific), lysates were injected (200 μl per injection) onto a Superose 6 10/300 GL column (GE Healthcare) equilibrated with PBS (pH 7.2) with a flow rate of 0.2 ml min^−1^. Twenty four fractions, each 200 μl in volume, were collected in a low protein binding 96-deep-well plate (Eppendorf). Approximate protein concentrations were estimated using the EZQ Protein Quantitation Kit (Thermo Fisher Scientific). Tris–HCl (1 M, pH 8.0) was added to each fraction to a final concentration of 0.1 M Tris–HCl to adjust the pH to 8.0. After reduction and alkylation using DTT and iodoacetamide, respectively, proteins in each fraction were digested to peptides for 18 h at 37 °C using either trypsin alone or both LysC & trypsin diluted in 0.1 M Tris–HCl (pH 8.0) at a final enzyme to protein ratio of 1:50 by weight. For peptide desalting, TFA was added to a 1% (v/v) final concentration, and peptides were purified using a Sep-Pak tC18 96-well *μ*-elution plate (Waters). Peptides were eluted with 500 μl of 50% (v/v) acetonitrile in 0.1% TFA, and dried in a SpeedVac prior to resuspension in 5% (v/v) formic acid. Peptide concentrations were determined using the CBQCA assay (Thermo Fisher Scientific) after 25-fold dilution of peptide samples in 0.1 M borate buffer (pH 9.3).

### LC-MS/MS and Analysis of Spectra

Using a Thermo Scientific Ultimate 3000 nanoHPLC system, an equal volume of digested peptides from each SEC fraction (approximately 10 μl, corresponding to a maximum of 1 μg peptide in the most abundant fraction) in 5% (v/v) formic acid was injected onto an Acclaim PepMap C18 nano-trap column (Thermo Fisher Scientific). After washing with 2% (v/v) acetonitrile in 0.1% (v/v) formic acid, peptides were resolved on a 150 mm × 75 μm Acclaim PepMap C18 reverse-phase analytical column over a gradient from 2% to 80% acetonitrile over 100 min with a flow rate of 300 nl min^−1^. The peptides were ionized by nano-electrospray ionization at 1.2 kV using a fused silica emitter with an internal diameter of 5 μm (New Objective). Tandem MS analysis was carried out on an LTQ Orbitrap Velos mass spectrometer (Thermo Fisher Scientific) using collision-induced dissociation fragmentation. Data-dependent acquisition (DDA) involved acquiring MS/MS spectra on the 30 most abundant ions at any point during the gradient. The raw MS proteomics data have been deposited to the ProteomeXchange Consortium (http://proteomecentral.proteomexchange.org/) *via* the PRIDE partner repository (35) with the dataset identifier PXD033459.

Raw data were processed using MaxQuant software (http://www.coxdocs.org/doku.php?id=maxquant:start, version 1.5.1.3) (36) using the default settings and searched against the human UniProt database (June 7, 2011 release) with common contaminant entries. The settings used for MaxQuant analysis were as follows: enzymes set as LysC/P and Trypsin/P, with maximum of two missed cleavages; fixed modification was carbamidomethyl (Cys); variable modifications were acetyl (protein N-term), carbamidomethyl (His, Lys), carbamidomethyl (N-term), deamidation (Gln, Asn), diCarbamidomethyl (His, Lys), diCarbamidomethyl (N-term), Gln to pyro-Gle, Oxidation (Met); mass tolerance 20 ppm (FTMS) and 0.5 Da (ITMS); false discovery rate (FDR) for both protein and peptide identification was 0.01. The ‘Re-quantify’ and ‘Match between runs’ features were both enabled. See supplemental Table S1 for the proteinGroups.txt output file from MaxQuant analysis. Samples generated by digestion with LysC+trypsin and trypsin alone were both analyzed together as identical technical replicates in the MaxQuant parameters file.

Note that initial attempts to analyze the 384 raw files with MaxQuant indicated errors in reading five files from the EXP2 samples (trypsin-digested fractions 17, 22, & 24 in the Control condition and LysC+trypsin-digested fractions 1 & 2 in the HSP90i condition). Therefore, for these fractions, only the sample digested with the other (MaxQuant-readable) enzyme schema was included in the MaxQuant input files.

### Initial SEC-MS Data Filtering and Exploration

All data filtering, exploration, and statistical analyses were performed using a combination of Microsoft Excel and *R* (https://cran.r-project.org/, version 3.6.2) with Tidyverse, unless otherwise stated. Specific *R* packages are referenced in the following text. The proteinGroups.txt file from the MaxQuant analysis (supplemental Table S1) was used as the input dataset for all downstream statistical analysis reported here. Of the 7401 entries in the proteinGroups.txt file, we filtered out 134 entries identified as potential contaminants, 91 as reverse matches, and 200 that were only identified by site. Additionally, 172 entries were removed as they were only identified by a single peptide. Of the remaining 6804 proteinGroups entries, we consolidated into single entries the splice variants, duplicated Entrez protein IDs, and duplicated HUGO Gene Names—resulting in 6427 unique protein entries for all subsequent analyses (supplemental Table S2). Gene Names that were not automatically assigned by MaxQuant were manually added from their Entrez protein IDs. To establish overlap and correlation between protein identifications across replicates, we used the *R* packages ‘UpSetR’ (37), ‘ggvenn’ (supplemental Fig. S1, *A* and *B*), and ‘heatmap.2’ (supplemental Fig. S1*C*). For scaled intensities in heatmaps and linegraphs, LFQ fraction intensities were scaled (using ‘resca’ function from *R* package ‘metan’ (38)) (supplemental Table S3). Scaling was performed per experiment (EXP1–EXP4) across all 48 fractions, such that the highest fraction intensity value for a protein in each EXP was set at 1, regardless of whether it was observed in the Control or HSP90i condition. For filtering based on number of replicates, we filtered for the proteins that had non-zero LFQ intensities in at least one of the 24 fractions (either Control or HSP90i condition—not necessarily both), in at least three of the four replicates, resulting in a list of 4645 proteins (supplemental Table S4). Heatmaps in the main figures were generated using the *R* packages ‘hclust’ for hierarchical clustering based on Euclidean distance with Ward-D2 linkage method and ‘heatmap.2’ for heatmap plotting. All boxplots, linegraphs, and Volcano plots were generated using the ‘ggplot2’ *R* package, unless otherwise stated.

### Limma-Based Differential Expression Analysis

All data shown at a total or summed level (*i.e.*, without individual fraction values) have been processed in the following way. Starting with the list of 4645 filtered proteins (supplemental Table S4), we added up all 24 LFQ intensities (F01:F24) for Control and HSP90i conditions separately. After log_2_-transforming and normalizing these data (variance-stabilizing normalization) (supplemental Fig. S1, *D* and *E*), we evaluated missing values, which were clearly biased towards proteins with lower LFQ intensities (supplemental Fig. S1*F*). Additionally, there were a larger number of missing values in Experiment 1 (both Control and HSP90i) (supplemental Fig. S1*G*). Based on these observations, we imputed the missing values using a manual left-censored Missing Not At Random method against a Gaussian distribution with a left-shift of 1.8 and a scale of 0.3. Using these parameters, we performed differential expression analysis using the *R* package 'Differential Enrichment Analysis of Proteomics Data' (DEP, https://bioconductor.org/packages/release/bioc/html/DEP.html) (39) on the contrast between HSP90i and Control, with differential proteins (DPs) set as those with an adjusted *p*-value of < 0.05 and an absolute log_2_FC threshold of 1 (*i.e.* Fold Change <−2 or >2). The adjusted *p*-values depicted in summed boxplots for all figures are based on differential expression values calculated by this workflow. To calculate enriched Gene Ontology (GO) terms, we separately entered the significantly downregulated and upregulated DPs into GOnet (40), using the full list of 4645 filtered proteins as the background. Networks were visualized using CytoScape (version 3.8.2). For individual fraction-level differential analysis of the filtered 4645 proteins, the same workflow was followed as for the summed protein intensities, except treating each of the 24 fractions as a separate Control *versus* HSP90i DEP analysis and without imputation of missing values. The DPs from each of the 24 fractions were combined, resulting in 366 DPs (supplemental Table S9). For the stringent fraction DPs (supplemental Table S10), only proteins that were DPs in two or more fractions were included. The network of stringent fraction DPs were generated using the Search Tool for the Retrieval of Interacting Genes (STRING) protein:protein interaction database (https://string-db.org/, version 11.5), with the following parameters: full STRING network type (both physical and functional interactions), edge thickness indicating strength of evidence for interaction, minimum required interaction score = 0.4 (medium confidence), Markov Clustering with inflation parameter = 2. GO term enrichment analysis was also performed on STRING, using the full list of 4645 filtered proteins as background. The canSAR curated interactome-based network was generated using the canSAR Protein Annotation Tool (https://cansarblack.icr.ac.uk/cpat). The canSAR interactome contains >1 million binary interactions for >19,000 human proteins. Interaction types are classified to reflect the method of experimental determination. A confidence level in the existence of a direct binary interaction is assigned in canSAR based on the type of the experiment and the number of independent publications reporting the interaction. All experimental determination types were included with a confidence level of ≥0.1.

### *CCprofiler* and PCprophet Protein Complex Detection

To identify the number of comprehensive resource of mammalian protein complexes (CORUM)-annotated protein complexes preserved in our dataset, we used either *CCprofiler* (28) or PCprophet (41), with the data frame of 6427 proteins used as a starting point. For *CCprofiler*, missing values in the computed list of protein traces were imputed by fitting a spline interpolation and normalized by cyclic loess (42). The proportion of intensities in assembled and monomer range were estimated using the *CCprofiler* ‘summarizeMassDistribution’ function. For feature detection purposes, protein traces were aggregated across conditions and replicates by summing up intensity values in each fraction. All subsequent complex-centric feature detection and coelution was performed against the default CORUM complexes reference data frame ‘corumComplexHypothesesRedundant’ in *CCprofiler*. Decoy complex queries were generated from this reference data frame (min_distance = 2), and protein complex features were detected from our normalized protein traces with the following parameters: corr_cutoff = 0.9, window_size = 5, rt_height = 1, smoothing_length = 5, collapse_method = “apex_network”, perturb_cutoff = 5%. The resultant complex features were filtered according to their apparent molecular weight (min_monomer_distance_factor = 1.2). The complex coelution peak groups with the largest number of coeluting protein subunits (‘getBestFeatures’ function) were then selected for statistical scoring at a 5% FDR. For PCprophet, data from supplemental Table S2 were separated into eight separate txt files (Ctrl_1–Ctrl_4, HSP90i_1–HSP90i_4), together with a sample ID key and calibration table for molecular weight estimation, as outlined in PCprophet instructions (https://github. com/anfoss/PCprophet/blob/master/PCprophet_instructions.md). PCprophet was run (using Python v3.7.3) on this dataset with default parameters against the CORUM database (using the ‘coreComplexes.txt’ file included in PCprophet), except with calibration by molecular weight (-cal) turned on, mapping of gene names to molecular weight (-mw_uniprot) included as a file from UniProt, and molecular weight–based complex collapsing (-co CAL flag). From the PCprophet output, the ‘DifferentialProteinReport.txt’ file was used to identify differential proteins with a ‘Probability_differential_abundance’ >0.5 (supplemental Table S7) and a combination of the ‘ComplexReport.txt’ and ‘DifferentialComplexReport.txt’ files to identify positive complexes (‘Is Complex’ = Positive) and differential complexes (‘Is Complex’ = Positive AND ‘Probability_differential_abundance’ >0.5) (supplemental Table S8). The SEC profiles of all subunits identified by PCprophet in different complexes were plotted as linegraphs. The protein:protein interaction networks were generated by importing ‘PPIReport.txt’ PCprophet output into CytoScape (colour = Control or HSP90i; edge width = count(‘Replicate’) grouped by Control or HSP90i), and differential proteins from supplemental Table S8 were mapped onto the network nodes.

### SEC-Immunoblotting

The cell culture, compound treatment, cell lysis, and SEC protocols used for SEC-MS were followed as closely as possible for validation by size-exclusion chromatography–coupled immunoblotting (SEC-IB). Five 15 cm dishes (80% confluent) of HT29 cells were treated with 62.5 nM tanespimycin (equivalent to 5 × GI_50_ for the cell line) or mock-treated with equivalent volume of DMSO. After 8 h, the cells were scraped on ice in 500 μl of ice-cold PBS containing cOmplete Protease Inhibitor Cocktail EDTA-free (Roche) and Phosphatase Inhibitor Cocktails 1 & 2 (Sigma). The collected cells were sonicated with a Branson-Tip Sonicator (high power, three cycles: 30 s on, 30 s off) at 4 °C and then centrifuged at 17,000*g* for 10 min at 4 °C. Samples were filtered through 0.45 μm Ultrafree-MC centrifugal filter units (Millipore) at 12,000*g* for 10 min. Bicinchoninic acid (BCA) protein assays (Pierce) were performed on the filtrates for protein quantification.

Using an ÄKTApurifier UPC 10 FPLC system (GE Healthcare), lysates in PBS with protease and phosphatase inhibitor cocktails were injected (500 μl per injection, corresponding to 1–3 mg total protein) onto a Superose 6 10/300 GL column (GE Life Sciences) equilibrated with PBS (pH 7.2) with a flow rate of 0.2 ml min^−1^. After 10 ml of void volume, 500 μl fractions were collected using a low protein binding 96-deep-well plate (Eppendorf). Fractions were aliquoted and stored at −80 °C before adding 3× Blue Loading Buffer (Cell Signaling Technologies) to 25 μl of each fraction and running on NuPAGE 4–12% Bis-Tris gels (Invitrogen). Following gel transfer onto nitrocellulose (Invitrogen), membranes were blocked in Tris-buffered saline (50 mM Tris–HCl (pH 7.5), 150 mM NaCl) with 1% Tween-20 (TBS-T) supplemented with 5% bovine serum albumin (BSA) (for HOP immunoblotting) or milk powder (for all other antibodies) for 1 h before incubating with the appropriate concentration of primary antibody diluted in TBS-T with BSA or milk powder overnight. Antibodies for HSP90α/β (sc-7947, 1:500), CDC37 (sc-5617, 1:500), and AHA1 (sc-50527, 1:500) were from Santa Cruz Biotechnology; p23 (ab92503, 1:10,000) and Anillin (ab99352, 1:2000) from Abcam; BAG3 (10599-1-AP, 1:1000) from Proteintech; Hop (#4464, 1:2000) from Cell Signaling Technologies; and HSP70/HSP72 (ADI-SPA-810-D, 1:2500) from Enzo Life Sciences. Membranes were then washed with TBS-T (3×, 5 min each) and incubated with horseradish peroxidase–conjugated secondary antibodies (GE Healthcare). Following another wash step in TBS-T, the horseradish peroxidase signal was detected by incubation with Pierce ECL Western Blotting Substrate (Thermo Fisher Scientific) and exposure to Hyperfilm ECL (GE Healthcare). Immunoblots shown are representative of three independent experiments.

For the HSP90 coimmunoprecipitation experiment, 1 mg of lysate from HT29 cells treated with tanespimycin or mock vehicle control as described above was diluted in modified RIPA buffer (50 mM Tris–HCl (pH 7.5), 150 mM NaCl, 1% IGEPAL CA-630, 0.5% sodium deoxycholate, 0.02% SDS, cOmplete Protease Inhibitor Cocktail EDTA-free (Roche)) to 190 μl final volume and incubated with 10 μl anti-HSP90–conjugated magnetic beads (clone SJ-90; LSBio catalog no. LS-C171164) for 1 h at 4 °C under rotary agitation. Two negative controls were incubated in parallel: one with a 50:50 mix of the tanespimycin and mock-treated HT29 lysates (1 mg total protein) in the presence of control magnetic beads without any conjugated antibody (LifeSensors, catalog no. UM400) (‘−IgG’), and a second control with the anti-HSP90 magnetic beads incubated with modified RIPA buffer only (‘+IgG’). Following five washes with modified RIPA buffer to remove unbound proteins, coimmunoprecipitated proteins were eluted from the magnetic beads by incubating with 10 μl 4× LDS Sample Buffer (Abcam) at 70 °C for 10 min, followed by transfer of the eluate into a separate tube and a further 10 min incubation at 70 °C in the presence of DTT (50 mM final concentration) to reduce the eluted proteins fully. Eluted proteins were loaded onto separate gels for each individual immunoblot. SDS-PAGE and immunoblotting were performed as described above, except with rabbit anti-HSP90 clone C45G5 (Cell Signaling Technologies #4877, 1:2000) for the HSP90 immunoblot and a 1:500 dilution (instead of 1:2000) for the Anillin immunoblot. See supplemental Fig. S9 for uncropped images of all immunoblots displayed in this article.

### Immunofluorescence

For Anillin immunofluorescence, HT29 cells were seeded onto 1% gelatin-coated coverslips (VWR 631-0152) in a 12-well plate. After 18 h, cells were treated with 62.5 nM tanespimycin for 8 h or 24 h, or the equivalent volume of DMSO vehicle control for 24 h. All subsequent fixation, permeabilization, and staining steps were performed at room temperature. Cells were fixed with 4% paraformaldehyde for 15 min and permeabilized with 0.1% Triton X-100 for 10 min. After blocking for 30 min in PBS with 1% BSA, cells were incubated with mouse anti-Anillin (Abcam ab211872, 1:150) for 2 h, followed by Alexa Fluor 647–labeled goat antimouse (Invitrogen A28181, 1:1000) for 1 h in the dark, both diluted in PBS with 0.1% BSA. Cells were stained for F-actin with Phalloidin-iFluor 488 (Abcam ab176753, 1:2000) in PBS with 1% BSA for 20 min in the dark. Cells were washed three times in PBS between each staining step. Finally, cells were stained with 0.1 μg/ml DAPI in PBS for 5 min in the dark, and coverslips were mounted onto glass microscope slides with ProLong Glass antifade mountant (Invitrogen P36980). Z-stack images were acquired on a Nikon A1R point-scanning confocal microscope with a 60× oil immersion objective and collapsed into 2D images using maximum intensity projection.

### Anillin Knockdown Experiments

siPools (siTools Biotech GmbH) comprising 30 siRNAs targeting Anillin (ANLN), Polo-like kinase 1 (PLK1), or no recognized mammalian gene (nontargeting control, NTC), or sterile water (untargeted control), were complexed for 15 min with DharmaFECT-4 transfection lipid (Horizon Discovery) in 75 μl/well OptiMEM (Invitrogen) at room temperature. Following the 15 min incubation, 2000 HT29 cells in 75 μl Dulbecco’s modified Eagle’s Medium (without penicillin or streptomycin) were added to each well, giving a final concentration of 25 nM siRNA and 0.8% lipid. Cells were incubated at 37 °C, 5% CO_2_ in an Incucyte Zoom (Sartorius AG) and scanned every 8 h for 2 days. After 2 days, 75 μl tanespimycin (final concentrations from 0.137–300 nM) or DMSO (0.1% final concentration) was added to each well. Cells continued to be scanned every 8 h for a further 4 days before being assessed for the number of viable cells using CellTiter-Blue (Promega) according to the manufacturer’s instructions. Cellular viability and confluency at each concentration of tanespimycin were calculated as a percentage of the DMSO control for the respective siRNA treatment with the dose–response curves plotted as nonlinear functions with variable slope in GraphPad Prism v.9.0.0 (GraphPad Software, https://www.graphpad.com/). The effect of siRNA knockdown on cells was calculated as a percentage of the NTC. All experiments were performed in biological triplicate.

### Isocitrate Dehydrogenase Activity Assays

Isocitrate dehydrogenase (IDH) activity was measured using the Isocitrate Dehydrogenase Activity Assay Kit (Sigma catalog no. MAK062) according to the manufacturer’s protocol. Briefly, one million HT29, HCT116, or BT474 cells were scraped on ice in 200 μl ice-cold IDH Assay Buffer (Sigma MAK062A) and centrifuged at 13,000*g* for 10 min at 4 °C. Fifty microliters of this cell lysate were added to an equal volume of the manufacturer’s Master Reaction Mix, with NAD^+^ or NADP^+^ for quantification of IDH3 or IDH1 & IDH2 activity, respectively. Absorbance readings at 450 nm for the samples compared with the NADH standard curve (always run in parallel with the samples) were used to estimate IDH activity.

## Results

### SEC-MS Provides a Platform to Investigate Changes in Protein Complex Distributions Following HSP90 Inhibition

**Fig. 1:**
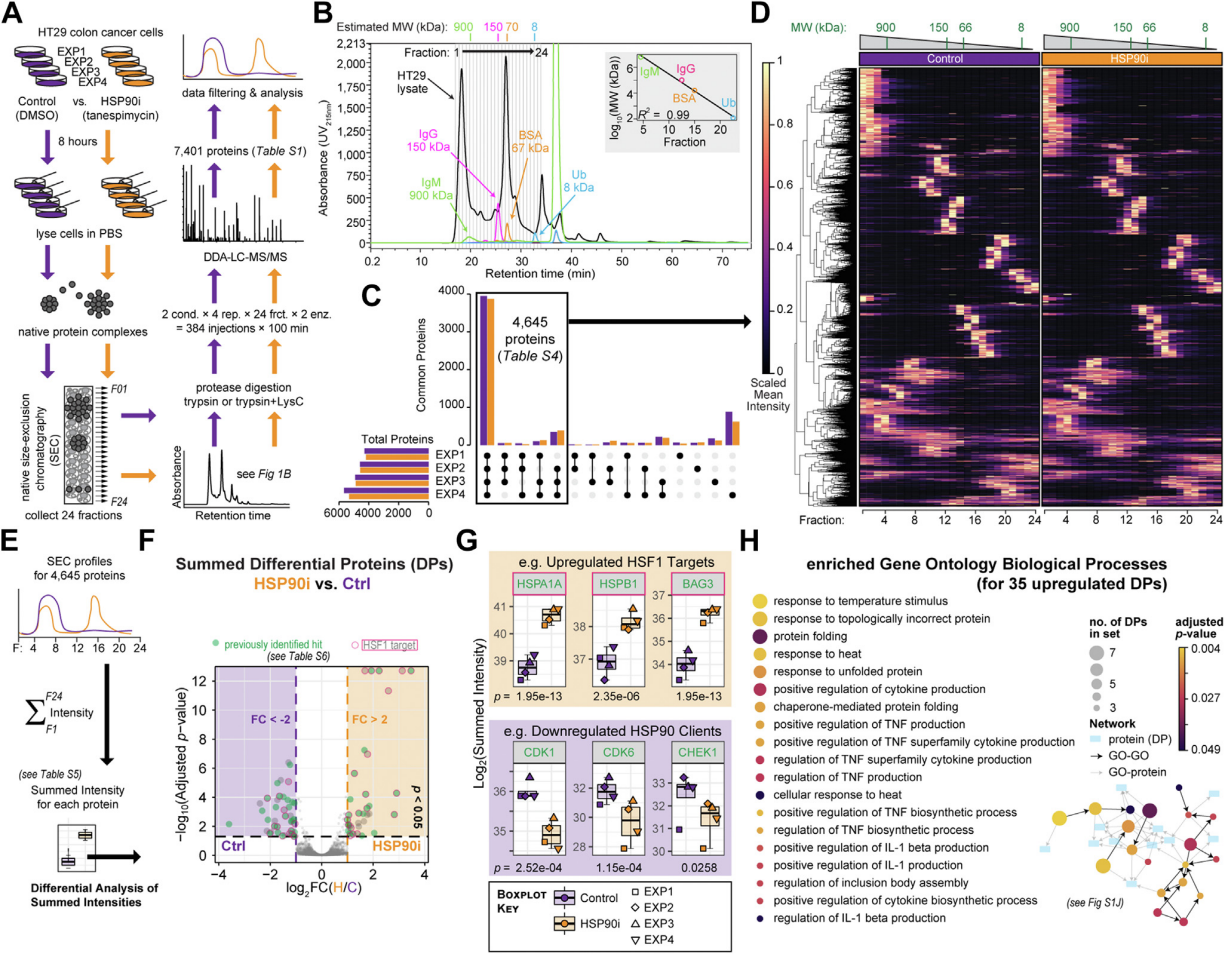
**SEC-MS approach to investigate changes in native protein complex distributions following HSP90 inhibition.***A*, workflow for SEC-based protein complex isolation and LC-MS/MS-based identification in HT29 colon cancer cells treated with five × GI_50_ (62.5 nM) of the HSP90 inhibitor tanespimycin or mock-treated with DMSO. *B*, UV chromatogram from one of the four control (DMSO-treated) biological replicates displaying the profile of the HT29 total cell lysate as it eluted from the Superose 6 SEC column across 24 fractions. The retention time (in minutes) and UV absorbance (at 215 nm) are represented on the *x*- and *y*-axes, respectively. Protein standards of known molecular weights (MWs) were injected onto the same column, and their elution peaks were used to estimate the MW range for each fraction using the *R* package *CCprofiler*. *C*, upset plot showing number of proteins identified in each of the four biological replicates (Control, *purple* and HSP90i, *orange*, represented separately). Same data depicted as Venn Diagrams in supplemental Fig S1, *A* and *B*. *D*, heatmap of scaled mean intensities for each of the 4645 proteins filtered in (*C*). Mean intensities across the four replicates were calculated for each protein by fraction (1–24) and condition (Control or HSP90i). Scaling was performed across all 48 fractions, such that the highest fraction intensity value for a protein was set at 1 regardless of whether it was observed in the Control or HSP90i condition. The dendogram cut-offs based on Euclidean distance matrix with the Ward-D2 linkage method are illustrated to the left of the heatmap. *E*, approach to calculate differential proteins (DPs) based on summed intensities across all 24 SEC fractions, using the *R* package DEP. See also supplemental Fig S1*I* and supplemental Table S5. *F*, volcano Plot calculated using the *R* package DEP, based on summed intensities for each of the 4645 filtered proteins. Log_2_-transformed Fold Changes (log_2_FC) and negative log_10_-transformed adjusted *p*-values (two-tailed Student’s *t* test with Benjamini-Hochberg correction) are plotted on the *x-* and *y*-axes, respectively. Proteins with *p* <0.05 and absolute log_2_FC >1 (*i.e*., absolute FC >2) are magnified. Proteins identified as ‘hits’ in previous high-throughput HSP90 proteomics studies are in *green*, and HSF1 targets are outlined in *magenta* (see also supplemental Table S6). *G*, summed intensities of known protein products of HSF1-activated genes (*orange*) and HSP90 clients (*purple*) are increased and decreased, respectively, following tanespimycin treatment. Box-and-whisker (Tukey) plots represent median, interquartile range, and absolute range for the four biological replicates. Adjusted *p*-values (Benjamini-Hochberg correction) calculated during differential expression analysis in (*E*) are indicated below each plot. *H*, Gene Ontology Biological Processes (GOBPs) significantly enriched among the 35 upregulated DPs identified in part (*F*) using GOnet, with the 4645 filtered proteins used as the background for the enrichment analysis. Details of proteins and GO terms in the network are included in supplemental Fig S1*J*. BSA, Bovine Serum Albumin; DEP, differential enrichment analysis of proteomics data; GO, gene ontology; HSP90, heat shock protein 90; IgM, Immunoglobulin M; IgG, Immunoglobulin G; MS, mass spectrometry; SEC-MS, size-exclusion chromatography coupled mass spectrometry; Ub, Ubiquitin.

We treated HT29 colon adenocarcinoma cells for 8 h with the HSP90 inhibitor tanespimycin (HSP90i), or mock-treated with DMSO vehicle (Control), before lysing in PBS without detergents or other chaotropes that could disrupt protein:protein interactions (Fig. 1*A*). At this tanespimycin exposure concentration and time, we had previously shown that client protein interactions with HSP90 co-chaperones (*e.g.*, CDC37, STIP1/HOP, AHA1) are disrupted, but major client protein degradation has yet to occur (31). Lysates from each of the four Control or HSP90i replicates (eight samples total) were fractionated by SEC to separate the protein complexes by molecular weight into 24 sequential fractions of equal volume (Fig. 1*B*). Each fraction was then subjected to standard protocols for bottom-up proteomics involving enzymatic digestion and LC-MS/MS, as described previously (29). To improve proteome coverage, we divided each fraction in two and digested one with a 1:1 mixture of the proteases LysC and trypsin, and the other with trypsin alone. Thus, we generated a total of 384 samples for LC-MS/MS analysis. The resulting raw data were analyzed using MaxQuant software (43). Overall, MaxQuant identified 111,365 peptides across 7401 proteinGroups (supplemental Table S1). After removing false identifications, and using a threshold of at least two peptides detected per protein at a FDR < 0.01, this equated to 6804 protein groups, representing 6427 unique proteins (following consolidation of duplicates into single entries) (supplemental Tables S2 and S3).

Our data show a strong overlap between the biological replicates, with 4645 proteins detected in three or more of the four experiments for at least one of the two experimental conditions (*i.e.*, Control or HSP90i) (Figs. 1*C*, S1, *A* and *B*). For these 4645 proteins (supplemental Table S4), there was a good pairwise correlation between the four replicates—both when comparing the summed LFQ intensities across all fractions, and when comparing LFQ intensities for each SEC fraction separately (supplemental Fig. S1*C*). Using a heatmap to visualize scaled mean intensities for each of the 4645 proteins across the 24 fractions (Fig. 1*D*), we did not notice drastic differences between the Control and HSP90i condition—suggesting that HSP90 inhibition does not trigger widespread remodeling of the native proteome under the conditions tested here.

### Summed Fraction Analysis Confirms Signature Proteomic Response to HSP90 Inhibition

In order to discount the possibility that the lack of obvious changes to the SEC-proteome between our two conditions was due to lack of target modulation (*i.e.*, HSP90 inhibition), we set out to confirm that tanespimycin treatment in our experiment led to the molecular changes expected in response to HSP90 inhibition. As our study is the first HSP90 inhibitor–based analysis of its kind, there were no other SEC-MS datasets for direct comparison. Therefore, we summed the individual intensities from all 24 fractions for the 4645 filtered proteins and performed differential expression analysis (supplemental Fig. S1, *D*–*H*) to identify proteins whose abundances changed significantly between the Control and HSP90i conditions (Fig. 1, *E*–*F*, supplemental Fig. S1*I*, and supplemental Tables S5 and S6)—in essence replicating previous bulk whole-proteome analyses of protein abundance changes in HSP90 inhibitor–treated cells (19, 20, 21, 22, 23, 44, 45, 46). Of the 76 DPs identified in our summed analysis (adjusted *p* < 0.05 and absolute log_2_(Fold Change) >1) (supplemental Fig. S1*I*), 47 DPs had also been identified in previous HSP90 inhibitor-proteomics studies (Figs. 1, *F*–*G* and S1*I*, in green; supplemental Table S6), including 27 of the 41 downregulated proteins. Furthermore, we confirmed induction of the heat shock response—triggered by activation of HSF1 following HSP90 inhibition (47)—as indicated by the presence of 20 HSF1 targets (48) among the 35 upregulated proteins (Fig. 1, *F*–*G* magenta outlines, supplemental Fig. S1*I* magenta stars, supplemental Table S6) and corresponding enriched Gene Ontology Biological Processes (40) such as ‘response to heat’, ‘response to unfolded protein’, and ‘chaperone mediated protein folding’ (Figs. 1*H* and S1*J*). In total, 58 of the 76 DPs here (76%) either had been identified in previous HSP90i proteomics datasets or were known HSF1 interactors. If we extended our comparisons to include all 113 significantly modulated proteins based on adjusted *p* < 0.05, this number now expanded to 89 proteins (79%). Therefore, we were confident that we had achieved HSP90 inhibition in our experiment.

### Clear Changes Detected in SEC-MS Profiles of Canonical HSP90 Inhibitor–Modulated Proteins

**Fig. 2:**
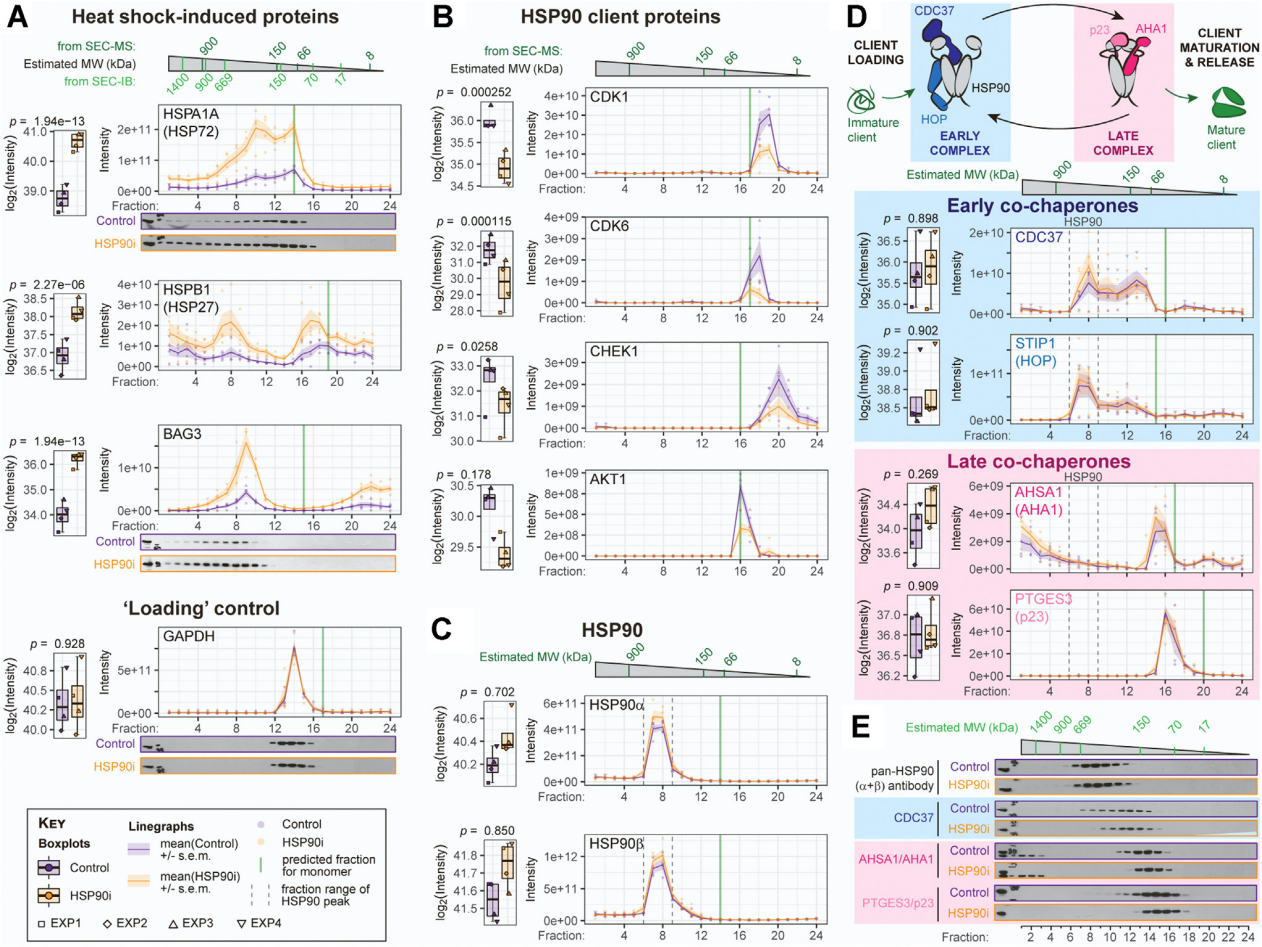
**Profiling changes in the HSP90 machinery.***A* and *B*, tanespimycin-induced induction of heat shock factor-1 (HSF1)–regulated proteins HSP70/HSP72 and BAG3 (*A*) and depletion of the HSP90 client proteins CDK1, CDK6, CHEK1, and AKT1 (*B*) observed in the SEC-MS dataset and confirmed by SEC-Immunoblot (SEC-IB). GAPDH levels were used as a ‘loading’ control. *C*, both inducible (HSP90AA1/HSP90α) and constitutive (HSP90AB1/HSP90β) cytoplasmic HSP90 isoforms elute predominantly between fractions 6 and 9 and do not change significantly following HSP90 inhibitor treatment. *D*, HSP90 co-chaperones considered to play a role early in HSP90’s ATP-dependent client maturation cycle (*blue* hues) coelute with HSP90, whereas later co-chaperones (*pink* hues) do not. See also supplemental Fig S2. *E*, SEC-IB profiles of the HSP90 machinery identifies subtle yet clear shifts of the co-chaperone CDC37 to lower molecular-weight fractions. All SEC-IBs are representative of three independent biological replicates. CDK, cyclin-dependent kinase; HSP90, heat shock protein 90; SEC-MS, size-exclusion chromatography–coupled mass spectrometry; SEC-IB, size-exclusion chromatography–coupled immunoblotting.

Having confirmed that the proteins whose overall summed abundances changed in our dataset were consistent with previous bulk proteomic studies of HSP90 inhibitor–treated cell lines, we proceeded with the aspect that was unique to our study: the fact that we had SEC traces for each individual protein. We first focused on the same two molecular hallmarks of HSP90 inhibitor treatment that we had evaluated with the summed differential protein analysis, that is, increased levels of the HSF1 target gene products HSP72, HSP27, and BAG3, and depletion of the HSP90 client proteins cyclin dependent kinase (CDK)1, CDK6, and CHEK1, as well as AKT1 (Figs. 2, *A* and *B* and S2*A*). In both cases, the SEC-MS profiles demonstrated clear and consistent trends in line with the summed analysis. Products of HSF1 target genes showed a general increase in all SEC fractions (Figs. 2*A* and S2*A*), whereas HSP90 clients generally eluted in one clear peak near the estimated molecular weight of the monomeric species, which had lower intensities in the HSP90i condition—consistent with client protein destabilization and degradation (Fig. 2*B*). Interestingly, we note that the profile of HSF1 itself did not differ appreciably between Control and HSP90i conditions (supplemental Fig. S2*A*), despite canonical models of heat shock response induction involving HSF1’s dissociation from HSP70 and/or HSP90 prior to trimerization, activation, and nuclear translocation (49, 50). It is possible that induction of HSF1 target gene-product expression—which we clearly observe here—requires release and nuclear translocation of only a minor fraction of the total HSF1 pool that is not easily detected with this SEC-MS workflow.

We validated the global SEC-MS data by repeating our HSP90 inhibitor treatment and initial SEC-fractionation, followed by SDS-PAGE and immunoblotting for individual proteins (SEC-IB). In the majority of cases, our targeted immunoblot analysis correlated well with the MS readout. For example, SEC-IB confirmed the increase across all fractions in HSP72 and BAG3, with the loading control GAPDH remaining unchanged (Fig. 2*A*).

### Distinct SEC Profiles and Minimal Changes Following HSP90 Inhibition for HSP90 Machinery Subunits

Switching focus to HSP90, the two major cytoplasmic HSP90 isoforms were distributed almost exclusively in fractions 6 to 9 for both SEC-MS and SEC-IB readouts (Fig. 2*C*)—consistent with a high molecular-weight oligomeric complex observed at 400 to 500 kDa by targeted native complex separation approaches (51, 52). Note that our HSP90 profiles indicate that a negligible fraction of the total HSP90α and HSP90β populations are present solely as dimers or monomers in HT29 cells, in agreement with higher-order HSP90-containing ‘epichaperome’ assemblies in cancer (15, 26). Similarly, the ER-resident HSP90 isoform HSP90B1/GRP94 was present almost exclusively in the higher molecular-weight fraction range, whereas the mitochondrial HSP90 TRAP1 mostly eluted as a monomer (supplemental Fig. S2*B*)—a finding that was also apparent when we plotted the SEC-MS profiles of these HSP90 isoforms from a HeLa-CCL2 dataset (53) (supplemental Fig. S2*C*). By contrast, the profiles of HSP72 and HSP27 had multiple peaks representing both monomeric and higher molecular-weight populations (Fig. 2*A*). Also in contrast with the other heat shock proteins, neither the profiles nor total abundance of the HSP90s changed between control and treated conditions (Figs. 2*C* and S2*B*)—a finding that is consistent with previous observations that HSP90 inhibition does not induce further HSP90 expression (19, 54, 55, 56).

HSP90 functions as part of a large multiprotein complex, consisting of dozens of co-chaperones that are required for various parts of its ATP-dependent client activation cycle (57). We had previously shown that the HSP90 co-chaperones HOP, CDC37, AHA1, and p23 all dissociate from HSP90:client protein complexes in HT29 cells under the same tanespimycin treatment conditions employed here (31). Therefore, we were surprised to find minimal changes to the SEC profiles of these co-chaperones following HSP90 inhibition (Figs. 2*D* and S2, *D* and *E*). Profiles of the co-chaperones could be grouped according to their role in HSP90’s ATP-driven client activation cycle: the early co-chaperones STIP1/HOP and CDC37, both involved in the loading of client proteins onto HSP90, had major peaks that overlapped with HSP90 (*i.e.*, fractions 6–9) (Fig. 2*D*). Later co-chaperones that are involved in client maturation and release (*e.g.*, AHA1, p23) did not cofractionate with HSP90 and were found in lower molecular weight fractions (Figs. 2*D* and S2*D*). Note that the protein phosphatase PPP5C/Ppt1, which dephosphorylates both HSP90 and CDC37 prior to ATP hydrolysis by the chaperone machinery (58, 59), also coeluted with the HSP90α/β peak (supplemental Fig. S2*D*). Our findings suggest that interactions between HSP90 and the late co-chaperones are not preserved through the sample preparation protocol, and perhaps that these interactions are weaker or more labile than those between HSP90 and the early co-chaperones. Interestingly, of the two main E3 ubiquitin ligases associated with the HSP90 machinery, CHIP/STUB1 cofractionated with HSP90, whereas CUL5 did not (supplemental Fig. S2*E*).

Following up the SEC-MS with SEC-IB analysis, we noticed subtle yet clear changes to the distribution of the early co-chaperone CDC37 following HSP90 inhibition, with the fraction distribution becoming narrower in the treated samples (Fig. 2*E*). We note that the tightening of the distribution was skewed towards the lower molecular weight fractions, suggesting that the higher molecular weight fractions—which overlapped with the HSP90 SEC-IB distribution—were disrupted following HSP90 inhibition, in line with our previous observations (31). Together with the lack of coelution of late co-chaperones with HSP90, these SEC-IB results suggest either that most of the co-chaperone population in both Control and HSP90i cells is not in complex with HSP90, or that the majority of HSP90:co-chaperone interactions are too labile to withstand sonication and fractionation, as required for the SEC protocol.

### Identification of Differential Protein Complexes Based on SEC Coelution Feature Detection

**Fig. 3:**
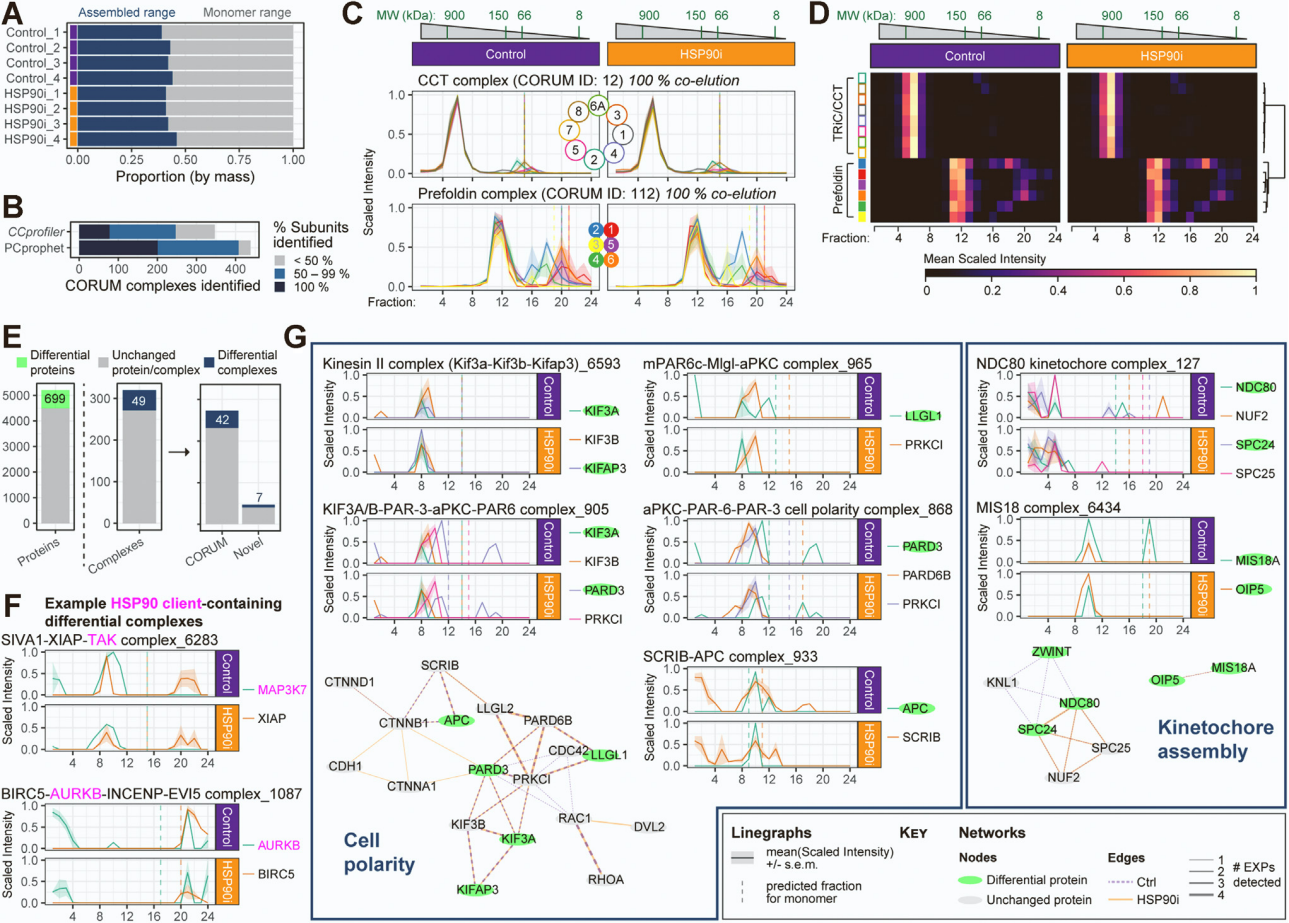
**Complex-centric analysis to identify CORUM protein complexes in SEC-MS dataset.***A*, global statistics of the proportion of the protein signal in each sample attributed to assembled or monomeric states in our dataset of 6427 proteins, as estimated by *CCprofiler*. *B*, number of CORUM-annotated protein complexes identified based on coelution of the SEC-MS profiles of their constituent subunits, using *CCprofiler* or PCprophet packages. The percentage of subunits identified are also indicated. *C*, mean scaled intensity profiles for each subunit of the hetero-oligomeric chaperones TRiC/CCT (*top*) and Prefoldin (*bottom*). Both complexes were fully detected by *CCprofiler*, but the higher-order chaperone consisting of Prefoldin and TRiC/CCT was not detected. *D*, heatmap of mean scaled intensities for TRiC/CCT and Prefoldin complex subunits. The dendogram cut-offs based on Euclidean distance matrix with the Ward-D2 linkage method are illustrated to the right of the heatmap. Similar data for the 26S proteasome are shown in supplemental Fig S3*C*. *E*, number of proteins (*left*) and protein complexes (*right*) identified by PCprophet as being differentially regulated following HSP90 inhibition. The protein complexes identified were further separated as those annotated on CORUM and those that were ‘novel’ complexes. See supplemental Tables S7 and S8 for full list of proteins and protein complexes, respectively, identified by PCprophet. *F*, mean scaled intensity profiles for each identified subunit of the differential protein complexes SIVA1–XIAP–TAK (CORUM ID: 6283) and BIRC5–AURKB–INCENP–EVI5 (CORUM ID: 1087). Established HSP90 clients are indicated in *magenta*. *G*, mean scaled intensity profiles (from supplemental Table S3) for selected differential protein complexes, grouped according to their biological function. Protein:protein interaction networks consisting of subunits within these complexes are also shown. *Green nodes* represent differential proteins identified using PCprophet’s protein-centric analysis. Edge width represents the number of experiments in which the interaction was confidently detected by PCprophet, with the edge color representing Control or HSP90i detections. Profiles of all 49 differential complexes are shown in supplemental Fig S4 and all protein:protein interactions networks identified by PCprophet in supplemental Fig S5. *Dashed vertical lines* on linegraphs indicate the fraction in which the monomer would be detected, based on the UniProt-annotated molecular weight. HSP90, heat shock protein 90; SEC-MS, size-exclusion chromatography–coupled mass spectrometry.

The observation that certain HSP90 complex subunits coeluted, whereas others did not, drove us to determine more globally the degree to which protein complexes were preserved in our dataset. According to the *R* package *CCprofiler*—developed to analyze SEC-MS data (28)—an estimated 39 to 46% of the protein mass was in the ‘assembled’ (vs. monomer) fraction size range in our complete dataset of 6427 proteins, with good consistency across all eight samples (Fig. 3*A*). The dataset contained 1796 of the 2532 proteins annotated in the CORUM protein complex database (60) (supplemental Fig. S3*A*), with 1457 of the 1753 CORUM-annotated protein complexes represented at 50% subunit coverage or more (supplemental Fig. S3*B*).

When assessing coeluting subunits from the SEC fraction profiles, *CCprofiler* identified 247 CORUM protein complexes, defined as at least 50% of the CORUM-annotated subunits classified as coeluting at 5% FDR (Fig. 3*B*). An alternative SEC-MS protein complex predictor, PCprophet (41), identified 408 CORUM complexes with the same threshold of at least 50% of subunits coeluting (Fig. 3*B*).

The discrepancy between protein complex coverage and coelution in our dataset—and, indeed, in all SEC-MS studies to date (28, 29, 32, 41, 53, 61)—is likely due to a number of factors. For one, not all CORUM-annotated complexes will be present in all cell types. Perhaps more importantly, the lysis conditions used—involving considerable sample processing time prior to SEC-based separation—will not preserve highly dynamic, transient, or labile interactions. To illustrate this point, the hetero-oligomeric chaperones TRiC/CCT and Prefoldin coeluted fully as individual protein complexes, but the higher-order chaperone CCT-Prefoldin (62) was not preserved (Fig. 3, *C* and *D*). Similarly, the majority of 20S proteasome core particle subunits eluted as a peak distinct from the 19S regulatory particle (supplemental Fig. S3*C*), suggesting that the 26S proteasome (63) is disrupted during sample processing.

The 245 complexes *CCprofiler* identified in our dataset is of a similar order of magnitude as other SEC-MS studies (supplemental Fig. S3*D*), although the higher numbers in those studies were presumably as a result of smoother elution profiles by SEC into more fractions and/or fewer missing values due to use of data-independent acquisition (DIA) for MS. Indeed, when Heusel *et al.* reanalyzed their SEC-fractionated HEK293 cell lysate with data-dependent acquisition MS (DDA-MS) instead of DIA-MS, they observed a >50% drop-off in CORUM complex identification (298 vs. 621) using the same *CCprofiler* parameters (28) (supplemental Fig. S3*D*).

Next, using PCprophet’s differential analysis workflow, we identified 699 proteins and 49 protein complexes as being significantly altered between our two conditions (Fig. 3*E* and supplemental Tables S7 and S8). As a percentage of the total positive IDs in the analysis, both the altered proteins (13.4%) and complexes (15.3%) were lower than that observed by Fossati *et al.* using the same PCprophet analysis workflow for comparing HeLa-CCL2 cells at interphase *versus* mitosis (approximately 30% and 26%, respectively) (41). This was not necessarily surprising, given that HSP90 is not a typical molecular chaperone responsible for general folding of the majority of the proteome (as opposed to HSC70, for example), but rather has a small subset of clients. Therefore, one would not expect the changes induced by HSP90 inhibition to be as widespread as the difference between two cell cycle states.

The 49 HSP90i-modulated protein complexes identified through PCprophet spanned diverse biological processes, consistent with the diverse functional nature of HSP90’s clientele (supplemental Fig. S4). Several of these protein complexes contained *bona fide* HSP90 clients, for example, the oncoprotein kinases TAK1/MAP3K7 (64, 65), Aurora kinase B (66, 67), and class 3 PI3-kinases PIK3C3 and PIK3R4 (68, 69), as well as their associated tumor suppressor Beclin1/BECN1 (70, 71) (Figs. 3*F* and S4). Furthermore, multiple signaling hubs known to require HSP90 activity were represented, including several protein complexes central to cell polarity (72, 73, 74) and kinetochore positioning during mitosis (75) (Fig. 3*G*). Importantly, not all of these HSP90i-modulated complexes are known direct HSP90 interactors. For example, neither of the HSP90i-modulated kinetochore-related complexes identified—MIS18 and NDC80—contain known HSP90 client proteins. Rather, HSP90 inhibition is proposed to impair kinetochore formation *via* destabilization and degradation of the MIS12 complex subunit DSN1 and/or the centromere-localized Polo-like kinase PLK1 (76, 77, 78)—neither of which belong to NDC80 or MIS18 complexes. The identification of such indirect HSP90-dependent complexes highlights the utility of our SEC-MS approach, as they would not have been identified using HSP90-interactomics, nor by bulk differential proteomics (as the total abundance of the constituent subunits did not change significantly upon HSP90 inhibition).

### Distinct HSP90 Inhibitor–Induced Protein Hits Identified by Summed *Versus* Individual Fraction Differential Analysis

**Fig. 4:**
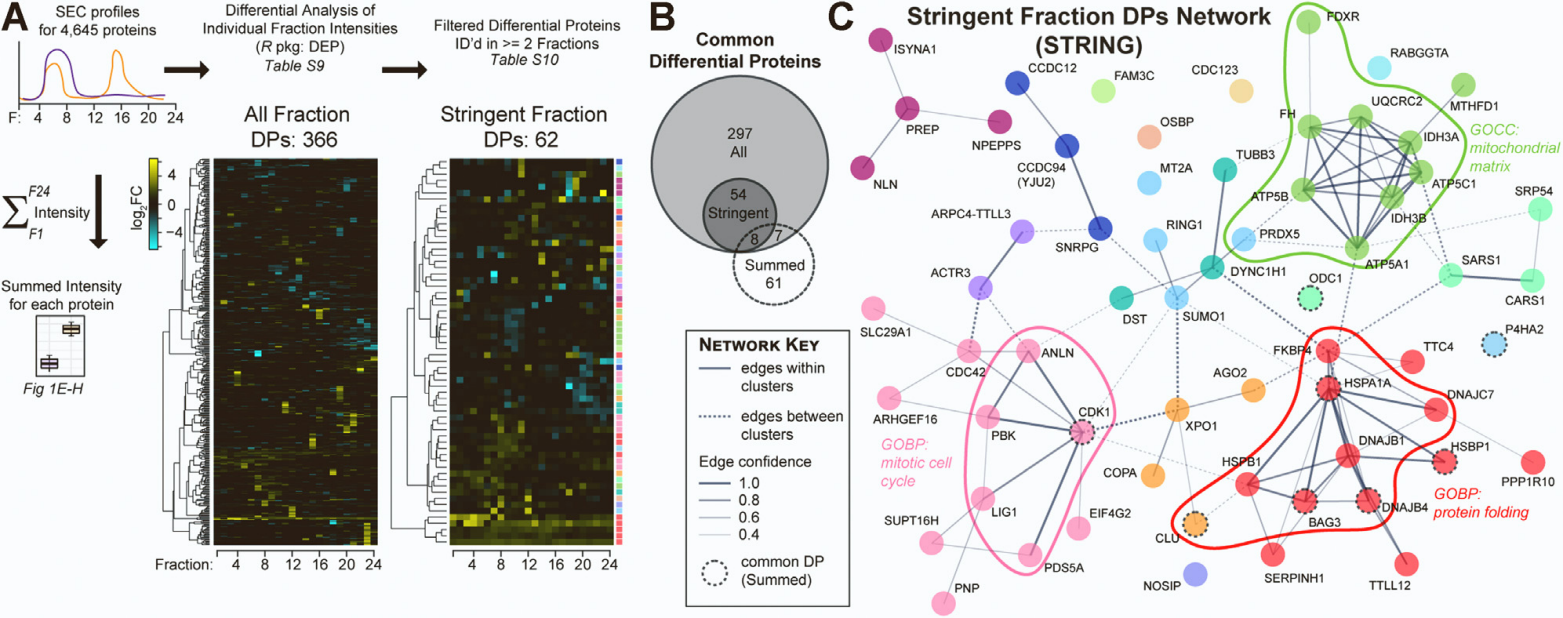
**Identifying proteins whose SEC profiles changed following HSP90 inhibition at an individual fraction level.***A*, approach to calculate DPs based on individual SEC fraction intensities between Control and HSP90i conditions, using the *R* package DEP. Heatmaps depict log_2_FC for the 366 proteins identified as hits (absolute log_2_FC >1 and adjusted *p*-value < 0.05) in any of the 24 SEC fractions (‘All Fraction DPs’, *left*) and those identified as DPs in two or more fractions (‘Stringent Fraction DPs’, *right*), using the *R* package DEP. Hits were clustered (Euclidean distance with Ward-D2 linkage) before plotting as heatmaps. Colors to the right of the Stringent Fraction DPs heatmap represent Markov clusters calculated on STRING-db with an inflation parameter of 2 (see also part (*C*)). *B*, Venn/Euler diagram (*R* package ‘eulerr’) representing the number of common proteins between the 366 All Fraction DPs and 62 Stringent Fraction DPs in (*A*) and 76 summed intensity–based DPs from Fig. 1. *C*, network generated by STRING protein:protein interaction database using the 62 Stringent Fraction DPs as the input. Colors represent clusters from Markov clustering (inflation parameter = 2). *Solid* and *dashed* lines indicate protein:protein interactions (edges) within and between clusters, respectively; weight of lines between nodes indicate strength of evidence for the interaction on STRING (both functional and physical protein associations). Proteins also identified *via* summed intensity analysis are depicted with *dashed circular* outlines. DEP, differential enrichment analysis of proteomics data; DP, differential protein; HSP90, heat shock protein 90.

Despite the identification of multiple protein complexes known to require HSP90 for their function (both directly and indirectly) using PCprophet’s differential analysis workflow, we were concerned that the limited number of total protein complexes positively identified would result in us missing important protein:protein interaction changes for proteins not assigned to a specific complex. Therefore, we complemented the automated PCprophet analysis by searching for differences between Control and HSP90i-treated samples at the SEC fraction level using the same differential analysis workflow we had used for the summed intensities in Figure 1, *E*–*H*, that is, treating our dataset as 24 different Control *versus* HSP90i comparisons (Fig. 4*A*).

We identified 366 unique DPs across all 24 fractions (Fig. 4*A* and supplemental Table S9). The majority of these proteins were only identified as DPs in one of the 24 fractions. Although single fraction changes could be indicative of important biological perturbations, we decided to filter out such singleton DPs in order to increase stringency and minimize the effect of stochastic fluctuations during SEC fractionation. After this step, we were left with 62 proteins that were DPs in two or more fractions (Figs. 4*A* and supplemental Table S10).

We were surprised to see only eight common proteins between the 62 stringent fraction DPs and the 76 summed DPs from Figure 1, *E*–*H* (Fig. 4*B*). Of these common proteins, all except one—CDK1, an HSP90 client—are known HSF1 target genes (48). Indeed, the only enriched GOBP term in the set of 62 stringent fraction DPs was ‘chaperone-mediated protein folding’ (adjusted *p* = 7.53e-7). This limited overlap provides further evidence that, with the exception of the most abundant heat shock proteins, our SEC-based approach identifies a distinct set of proteins compared to those that would be identified using total cell lysate–based comparative proteomics.

Next, we generated protein:protein interaction networks for the 62 stringent DPs using either the STRING protein:protein interaction database (79) or the curated interactome in the canSAR database (80) (Figs. 4*C* and S6). Both tools yielded networks with significantly more edges than expected by chance, that is, 47 edges expected by chance; 99 edges between 54 nodes observed with STRING (*p* = 3.25e-11); 76 edges between 43 nodes observed with canSAR. These highly connected networks suggested that the 62 DPs as a group were biologically related. STRING’s built-in Markov Cluster Algorithm (81) identified three large clusters (those with >five nodes each); the largest of these clusters (Fig. 4*C*, red nodes) was heavily enriched in molecular chaperones and co-chaperones (10 of the 12 cluster nodes) and included four of the eight DPs common with the summed DPs (Fig. 4*C*, dashed node outlines). Another cluster contained numerous cell cycle and cytoskeletal proteins (Fig. 4*C*, pink nodes). The final large cluster consisted of nine mitochondrial proteins (eight from the mitochondrial matrix), including members of the F_1_F_0_-ATP synthase F_1_ subcomplex (3/5 subunits) and IDH3 complex (2/3 subunits) (Fig. 4*C*, light green nodes).

### The Actin-Binding Oncoprotein Anillin is Recruited to Inhibited HSP90 Complexes

As the largest cluster from our Stringent Fraction DP network (Fig. 4*C*, red nodes) consisted exclusively of well-characterized heat shock response proteins, we decided to focus on the other two clusters to gain potentially novel biological insight. Of the four highest-confidence interactions within the cell cycle and cytoskeletal protein cluster (STRING edge confidence >0.9), CDK1, PBK, and PDS5A were previously identified as HSP90 inhibitor–modulated proteins (23, 25). We therefore probed further into the fourth node in this high-confidence edge network: ANLN/Anillin, a cytoskeletal scaffold protein that links RhoA, actin, and myosin during cytokinesis, with additional emerging nuclear roles in cancer-associated gene transcription (82).

**Fig. 5:**
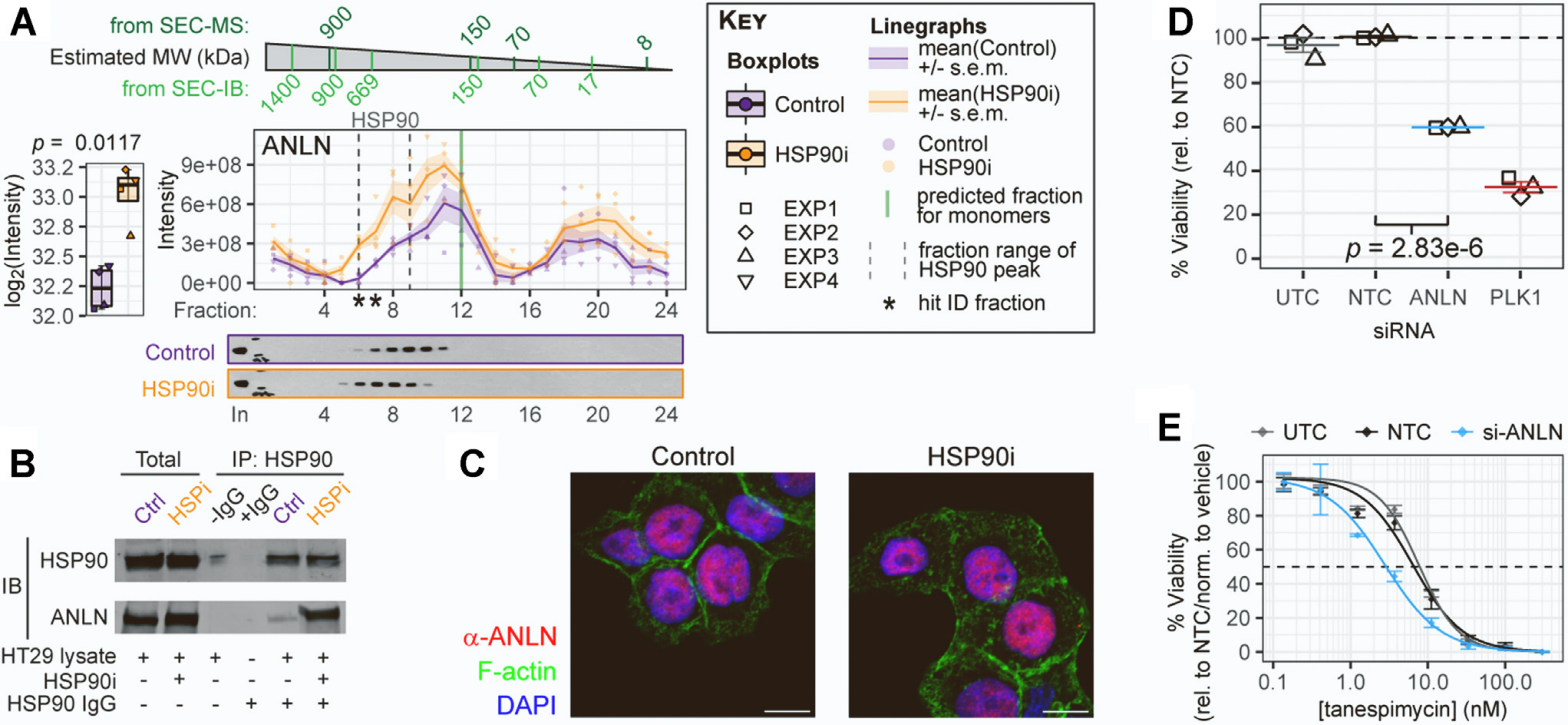
**Actin-binding protein Anillin/ANLN is recruited to inhibited HSP90 complexes.***A*, left, total ANLN/Anillin protein levels increase following HSP90 tanespimycin treatment (*p* = 0.0017, log_2_FC = 0.86). *Right*, SEC-MS and SEC-IB elution profiles indicate a shift towards higher molecular weight complexes for the major Anillin peak following HSP90 inhibition. This includes an increase in the same fractions as the HSP90 elution peak (linegraph, *dashed lines*). *B*, Anillin coimmunoprecipitation (IP) with HSP90 is increased following tanespimycin treatment. HT29 human colon cancer cells were treated with five × GI_50_ (62.5 nM) tanespimycin or mock-treated with the same volume of vehicle DMSO control, for 8 h. HSP90 IP was performed on 1 mg of lysate protein from each condition, and the resultant immunoblots (IBs) were probed for HSP90 and Anillin, as indicated. Fifteen microgram of lysate protein was loaded for the ‘Total’ lanes. *C*, Anillin remains predominantly nuclear upon HSP90 inhibition. HT29 cells were treated for 24 h with 62.5 nM tanespimycin or equal volume of DMSO vehicle control, before fixing, permeabilizing, and staining for Anillin, F-actin (phalloidin), and DNA (DAPI). Images represent maximum intensity projections from collapsed z-stacks. Scale bars represent 10 μm. See supplemental Fig S7*A* for expanded experiment data. *D* and *E*, knockdown of Anillin reduces HT29 cell viability and results in a two-fold sensitization to tanespimycin. HT29 cells were treated for 48 h with siTOOLs pool of 30 siRNAs (25 nM total concentration) targeted against Anillin (ANLN), nontargeting control NTC, untargeted control UTC, or death-inducing control PLK1, followed by treatment for 96 h with a range of tanespimycin concentrations (except for PLK1) or mock-treatment with vehicle control (0.1% DMSO), while still in the presence of the original siRNAs. Confluency was monitored every 8 h throughout the time-course by Incucyte (see supplemental Fig S7). Cell viability at the end of the time-course (144 h total) was measured using CellTiter-Blue cell viability assay. Cell viability results for the mock vehicle-treated controls only are plotted in (*D*), with adjusted *p*-value (as determined by one-way ANOVA with Tukey’s multiple comparison test) for Anillin *versus* NTC. Lines and error bars represent mean ± standard error for each condition from three biological replicates (EXP1–EXP3, different shapes). Plotting all the dose-response data (*E*) identified two-fold sensitization to tanespimycin treatment on Anillin knockdown in HT29 cells, with GI_50_ reduced to 2.9 nM (vs. 6.6 nM for NTC). Points and error bars represent mean ± standard error for each condition. Dose-response curve fitting was performed using the ‘Log[Inhibitor] *versus* normalized response – Variable slope’ non-linear regression model in Graphpad Prism. See supplemental Fig S7*D* for dose-response curves relative to vehicle-treated (0.1% DMSO) NTC control. HSP90, heat shock protein 90; SEC-MS, size-exclusion chromatography–coupled mass spectrometry; SEC-IB, size-exclusion chromatography–coupled immunoblotting.

Anillin is an important regulator of the Epithelial-to-Mesenchymal Transition during malignancy (82, 83), and is upregulated in a variety of tumors—including colon cancer (84, 85, 86, 87). Here, Anillin almost met our hit criteria in the initial global analysis of summed intensity changes (Fig. 1, *E*–*H*), missing out because it fell just under the fold-change threshold (Fig. 5*A* boxplot, *p* = 0.0117, log_2_FC = 0.86). The degree of Anillin modulation was strikingly consistent with the HSP90i-induced Anillin changes observed when we analyzed previous proteomics datasets in HeLa cervical (*p* = 0.0002, log_2_FC = 0.94), MDA-MB-231 triple-negative breast (*p* = 0.0411, log_2_FC = 0.92), and CAL-27 oral squamous (*p* = 0.0007, log_2_FC = 1.28) carcinoma cell lines (21, 23). Using our individual fraction-based approach, Anillin was a hit in fractions 6 and 7 (Fig. 5*A* linegraph, asterisks). Although Anillin’s SEC profile at first glance looked typical of HSF1-induced proteins, we noticed a subtle shift in the major peak towards higher molecular weight fractions in the HSP90i conditions (Fig. 5*A*, linegraph), as opposed to a global increase across almost all fractions (*e.g.*, compared with HSP72, HSP27, and BAG3 SEC profiles in Fig. 2*A*). This shift was even more apparent with an SEC-IB readout (Fig. 5*A*, immunoblots). As this shift resulted in more of the Anillin population overlapping with the HSP90 peak (Fig. 5*A*, linegraph, dashed lines), it occurred to us that Anillin might be interacting with HSP90 complexes and that this interaction was increased upon HSP90 inhibition. Indeed, immunoprecipitation of endogenous HSP90 confirmed that Anillin is recruited to HSP90-containing complexes following tanespimycin treatment (Fig. 5*B*). We did not observe any change upon tanespimycin treatment in Anillin’s predominantly nuclear localization (Figs. 5*C* and S7*A*), perhaps suggesting Anillin interacts with the HSP90 subpopulation in the nucleus.

We hypothesized that recruitment of Anillin to inhibited HSP90 complexes may be an important part of the cellular response to HSP90 inhibition. Knockdown of Anillin by pooled siRNAs for six days resulted in a 42% and 49% reduction in HT29 cell viability and confluency, respectively, *versus* the negative NTC (Figs. 5*D*, S7, *B* and *C*). Although this reduction in cell viability made it difficult to assess any further effect of Anillin knockdown on HSP90 inhibitor sensitivity (supplemental Fig. S7*D*), we identified a two-fold reduction in tanespimycin GI_50_ concentration to 2.9 nM (vs. 6.6 nM for the negative NTC siRNAs) (Fig. 5*E*). These findings lend further weight to the role of Anillin in malignancy (82, 88, 89) and suggest effects on HSP90-mediated networks as at least a partial mechanism for its pro-tumor function.

### Mitochondrial Matrix-Enriched Cluster Identifies IDH3 as a Novel HSP90-Dependent Protein Complex

**Fig. 6:**
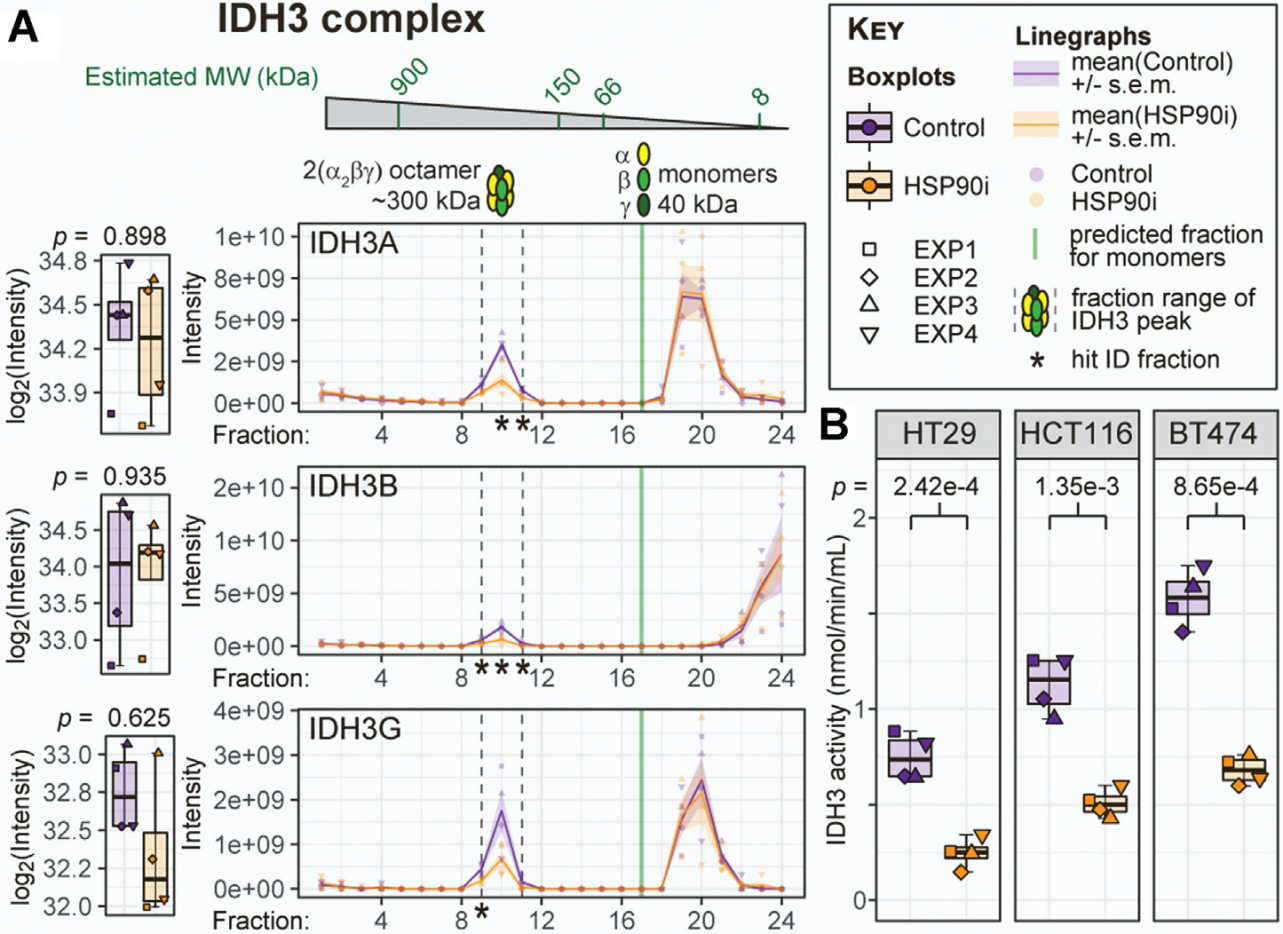
**Mitochondrial isocitrate dehydrogenase 3 complex is disrupted upon HSP90 inhibition.***A*, left, Box/Tukey plots illustrate that total levels of the three IDH3 protein complex subunits do not change following HSP90 inhibition. *Right*, SEC profiles identify a high molecular-weight peak (corresponding to the size of the octameric IDH3 complex) that is significantly reduced for all three subunits in HSP90 inhibitor-treated cells. *Asterisks* depict significantly differential fractions. *B*, IDH3 activity is significantly reduced upon HSP90 inhibition in HT29 human colon adenocarcinoma, HCT116 human colon carcinoma, and BT474 human breast ductal carcinoma cell lines. Activity was measured using the IDH Activity Assay Kit (Sigma) according to manufacturer’s instructions, using NAD^+^ as the cofactor. See supplemental Fig S8*B* for the corresponding assay with NADP^+^ as the cofactor, for estimating IDH1 and IDH2 activity. Adjusted *p*-values (two-tailed Student’s *t* test) are shown. HSP90, heat shock protein 90; IDH, Isocitrate dehydrogenase.

Finally, switching focus to the mitochondrial matrix–enriched cluster from our Stringent DP Network (Fig. 4*C*), we were especially interested in subunits mapping to two protein complexes with central roles in mitochondrial metabolism: the F_1_F_0_-ATP synthase complex V, and IDH3 (Figs. 6 and S8). The F_1_F_0_-ATP synthase complex has been reported previously to interact with HSP90 in human cancer cells, and disrupting this interaction results in degradation of a subset of HSP90 client proteins (90, 91). In the budding yeast *Saccharomyces cerevisiae*, several targeted and global approaches have identified genetic interactions between the yeast HSP90s Hsc82/Hsp82 and the ATP synthase complex F_1_ subunits Atp1, Atp2, and Atp3 (92, 93, 94). Strikingly, the equivalent subunits in human ATP synthase—ATP5F1A, ATP5F1B, and ATP5F1C—were all identified as Stringent DPs in this study (Figs. 4*C* and S8*A*). We found that all three subunits coeluted in a clear peak at the predicted molecular weight of this subcomplex, which was almost entirely reduced to baseline levels in the HSP90i condition (supplemental Fig. S8*A*). By contrast, no subunits of the peripheral stalk complex of ATP synthase coeluted at the predicted molecular weight of any subcomplexes (or of the fully-formed F_1_F_0_-ATP synthase complex, ∼592 kDa).

Whereas there was existing evidence of interaction between the ATP synthase F_1_ subcomplex and HSP90, we could not find any such evidence linking HSP90 with IDH3. Two of the three subunits of this complex were identified as Stringent DPs, and the third subunit—IDH3G—was identified in our less stringent list of 366 DPs at the individual fraction level. The total levels of none of the three subunits were changed (*p* > 0.05 and absolute log_2_FC <1) (Fig. 6*A*, boxplots). Therefore, the IDH3 complex would not be identified as a hit through traditional bulk proteomics. Plotting the SEC-MS traces, however, brought to light clear changes in a certain sub-population of IDH3 subunits in HSP90i-treated cells (Fig. 6*A*, linegraphs). The bulk of the signal for all three subunits was in low molecular-weight fractions, most likely representing the monomeric species. These peaks were the same in control and HSP90 inhibitor-treated conditions. However, a minor peak around fraction 10—representing a larger molecular-weight complex of approximately 300 kDa—was clearly reduced following HSP90 inhibition for all three subunits. This would be consistent with the size of an octameric complex, thought to represent the active form of IDH3 in cells (95).

We reasoned that HSP90 could play an important role in the maintenance of active IDH3 complexes. We showed using a cell-based reporter assay that IDH3 activity is reduced by >50% following HSP90 inhibition in HT29 cells, as well as in two other cell lines—HCT116 colon carcinoma, and BT474 breast ductal carcinoma (Fig. 6*B*). The effect was specific to this IDH complex, as the activities of IDH1 and IDH2—which use NADP^+^ as a cofactor, rather than NAD^+^—were unaffected (supplemental Fig. S8*B*-i). Finally, we confirmed that this was a general effect of HSP90 inhibition by performing the same assay in cells treated with two other HSP90 inhibitor chemotypes in HCT116 cells (supplemental Fig. S8*B*-ii). We therefore identify the IDH3 complex as a novel component of the HSP90-dependent proteome that would most likely have remained uncharacterized using traditional bulk proteomics or interactomics approaches.

## Discussion

Defining the scope of the HSP90-dependent proteome has been a subject of enquiry in both basic and translational biology for over a decade (18, 19, 20, 96, 97, 98). Here, we present an alternative approach to expand insight into this challenge, employing changes in the native complex profiles of the proteome rather than relying on changes in bulk abundance or direct interacting partners of HSP90. After confirming target engagement (*i.e.*, HSP90 inhibition) in our dataset through summed differential protein analysis (Figs. 1 and S1), we discovered through plotting SEC profiles of the HSP90 machinery that certain interactions were preserved, whereas others were almost entirely lost in these experiments (*e.g.*, HSP90 interactions with early vs. late co-chaperones) (Figs. 2 and S2). This ‘all-or-nothing’ phenomenon also held true at a global level: numerous obligate hetero-oligomeric protein complexes (*e.g.*, TRiC/CCT and Prefoldin; 20S and 19S proteasome) coeluted, whereas larger assembled complexes (*e.g.*, CCT-Prefoldin; 26S proteasome) did not (Figs. 3, *C* and *D* and S3*C*). Employing a complex-centric peak coelution approach through the packages *CCprofiler* and PCprophet, we identified 49 HSP90i-modulated protein complexes between the Control and HSP90i condition (Figs. 3, *E*–*G* and S4). These included several protein complexes containing known HSP90 clients, as well as protein complexes involved in downstream HSP90-dependent biological processes. To complement this complex-centric approach, we performed differential expression analysis at an individual fraction level, yielding 62 stringent DPs (Fig. 4). Finally, we validated two novel hits from these stringent fraction-level DPs—Anillin (Figs. 5 and S7) and the IDH3 complex (Figs. 6 and S8)—identifying these as potentially important components of the HSP90-dependent proteome.

Given our previous findings that HSP90:client:co-chaperone coimmunoprecipitates were disrupted in this cell line under identical tanespimycin treatment conditions to those employed in this study (31), we were surprised to detect minimal differences in the SEC profiles of HSP90’s co-chaperones between Control and HSP90i conditions (Figs. 2, *D* and *E* and S2*E*). This discrepancy is likely related to the fact that a considerable proportion of the co-chaperone populations did not coelute with the HSP90-containing fractions and instead eluted in fractions close to their predicted monomer weights. It is possible that the multiple handling steps required for the SEC workflow leads to loss of the more labile or dynamic interactions—as would be the case for co-chaperones, whose binding and release is dynamically regulated and highly dependent on the conformational and nucleotide-binding status of HSP90 (57). Alternatively, it has been proposed that HSP90 spends most of its dwell time in its open, ATP-bound conformation, in complex with early co-chaperones and primed for client protein loading (99). Therefore, ATP-competitive HSP90 inhibitors such as tanespimycin may exert their effects through a minor (albeit functionally critical) proportion of total HSP90 complexes within a cell. Our finding that only early co-chaperones have an SEC peak coeluting with HSP90 (Fig. 2, *D*–*E*) is consistent with this hypothesis.

Beyond the HSP90:client:co-chaperone complexes, our approach provides several novel insights into the HSP90 inhibition response that have so far remained uncharacterized through traditional comparative proteomics (*e.g.*, MS of control vs. treated whole cell lysates) or interactomics (*e.g.*, MS of HSP90 or co-chaperone coimmunoprecipitates; LUMIER and other bait:prey interaction screens). This was perhaps best exemplified by the limited overlap between DPs identified with our individual fraction approach and those identified for summed intensities (Fig. 4 vs. Fig. 1, *E*–*H*)—especially as the same workflow was used in both analyses. We demonstrate the utility of our dataset by identifying two as yet uncharacterized components of the HSP90-dependent proteome that were only identified as DPs with our individual fraction-level analysis.

We discovered that Anillin abundance was significantly altered in previous HSP90 inhibition datasets (21, 23); yet it had not, to our knowledge, been validated in these or any other HSP90-related studies. We followed up this DP for two main reasons. In the context of the present study, Anillin’s shift to higher molecular weight fractions upon HSP90 inhibition was a relatively rare trait in the dataset. We therefore reasoned that Anillin might be recruited to specific complexes upon HSP90 inhibition—and even that this recruitment might occur at the level of HSP90 complexes directly, given the significant increase by fraction-level differential analysis in the HSP90 elution peak (Fig. 5*A*). This was confirmed by coimmunoprecipitation of Anillin with HSP90, which increased following tanespimycin treatment (Fig. 5*B*). The second reason for our interest in Anillin was a growing body of research showing its role in tumor progression, across a range of indications (82, 83, 84, 85, 86, 87, 88, 100). Our finding that Anillin knockdown for six days results in a smaller number of viable cells (Fig. 5*D*) is consistent with this protein’s central role in cytokinesis (101). Although the effect of Anillin knockdown on sensitivity to tanespimycin was relatively modest (Fig. 5*E*), it suggests a potential relationship between HSP90 inhibitor efficacy and Anillin levels—which, as we have discussed, is overexpressed in several cancers. Anillin provides yet another potential link between the HSP90 machinery and cancer cell invasion, together with the cell polarity and kinetochore assembly–associated protein complexes identified as HSP90i-modulated by PCprophet in this study (Fig. 3*G*). We should note that the lack of obvious changes to the nuclear localization of Anillin following tanespimycin treatment (Figs. 5*C* and S7*A*) could indicate that HSP90 inhibition perturbs Anillin’s role in cancer-associated gene transcription, rather than its cytoskeletal scaffolding function (82). Given that increased nuclear (rather than cytoplasmic) levels of Anillin correlate with poorer outcomes in a range of cancers (82, 85), it is possible that nuclear Anillin:HSP90 complexes have an important biological impact on cancer cell survival.

Whereas careful mining of previous HSP90 inhibition proteomics datasets might have identified Anillin as an HSP90-modulated protein, the mitochondrial IDH3 complex has not been associated with the HSP90 inhibition response—and likely would have remained uncharacterized in this context through targeted interaction or differential abundance approaches. Importantly, we found that HSP90 inhibition specifically impaired activity of the IDH3 complex and not of the other two IDH family members IDH1 and IDH2 (Figs. 6*B* and S8*B*). IDH3 is not as well characterized as IDH1 and IDH2 in the context of cancer and other diseases (102); however, more recent studies implicate aberrant expression of IDH3—especially the alpha subunit—in malignancy (103, 104, 105). This is likely related to the fact that rewiring of energy metabolism is an extended hallmark of cancer (106). Indeed, the Warburg effect—where cells switch from mitochondrial oxidative phosphorylation to glycolysis as their predominant means of ATP production, even under oxygen-rich conditions—is commonly observed during the malignant process. As part of the tricarboxylic acid cycle, IDH3 would play a key role only in ATP production in cells that have not undergone Warburg-like metabolic transformations. Future studies could test whether the degree of glycolytic shift of a tumor correlates negatively with its sensitivity to HSP90 inhibitors. Furthermore, the fact that healthy cells are presumably more dependent on the tricarboxylic acid cycle—and therefore IDH3—than cancer cells might contribute to a narrowing of the therapeutic window for HSP90 inhibitors *in vivo*. More recent data suggest that IDH3 levels decrease during senescence (107)—a finding of potential importance in light of the interest in HSP90 inhibitors for targeting senescent cells (11).

The mechanism underpinning tanespimycin’s effects on mitochondrial matrix proteins (9/62 Stringent DPs in this study) remains controversial. There are conflicting data over the extent to which tanespimycin can penetrate mitochondrial membranes, and thus inhibit the mitochondrial HSP90 TRAP1, in cancer cells (108, 109). One study showed that tanespimycin affected TRAP1’s modulation of another mitochondrial matrix protein complex: succinate dehydrogenase, which functions in both the electron transport chain and TCA cycle (109). Alternatively, tanespimycin’s mitochondrial effects could be explained by the established role for cytoplasmic HSP90 in the *de novo* folding and import of mitochondrial preproteins (110, 111). At least in the case of F_1_F_0_-ATP synthase—of which we identified the three major F_1_ complex subunits as Stringent DPs—there is evidence to support physical interaction with cytoplasmic HSP90 in cancer cells (90, 91). Furthermore, global studies in budding yeast have revealed genetic interactions of the yeast ATP5F1A, ATP5F1B, and ATP5F1C orthologs and HSP90 (92, 93), potentially *via* a cytoplasmic co-chaperone with mitochondrial client–specific functions (94). Regardless of the exact mechanism or mechanisms at play, our work further adds to the evidence that HSP90 inhibition substantially impacts mitochondrial protein homeostasis and metabolism.

Despite the novel biology revealed in this study, several limitations of SEC-MS—as well as potential improvements—became apparent during our analysis. Almost 1500 CORUM protein complexes were detected at greater than 50% subunit coverage (supplemental Fig. S3*B*), yet *CCprofiler* and PCprophet assigned only 245 and 408 CORUM complexes, respectively, as having coeluting SEC traces (Fig. 3*B*). This number was slightly lower than existing published SEC-MS studies (supplemental Fig. S3*E*), which could be caused by a number of technical and/or biological differences in our dataset. For example, at the biological level, HT29 colon carcinoma cells could have fewer or more labile protein complexes than HEK293 and HeLa cells—two workhorse cell lines with wide-ranging abnormalities at the genetic and protein level (112, 113). At the technical level, a key component of unbiased complex-centric proteome profiling algorithms such as those implemented by *CCprofiler* and PCprophet is the consistent identification of the same set of target proteins in consecutive SEC fractions (28). Coelution feature detection is therefore greatly diminished by missing values: a common problem in label-free DDA-MS, which relies on stochastic peptide sampling and MS/MS fragmentation of only the top ‘n’ peptides or other ions in a specified mass-to-charge ratio window (114, 115). Missing values are substantially reduced using DIA workflows such as sequential window acquisition of all theoretical mass spectra (SWATH-MS) (116, 117), for which both *CCprofiler* and PCprophet were primary developed. Further factors limiting protein complex identification in our dataset (compared with previous datasets benchmarked by *CCprofiler* and PCprophet) include separation of the cell lysate into a smaller number of SEC fractions and the use of protein-level rather than peptide-level intensity data. Although we could have used the peptide-level intensities from MaxQuant for *CCprofiler* and PCprophet, we decided to avoid this approach in light of recent findings that peptide-level quantification results in significantly lower true positive rates than protein-level quantification for label-free DDA-MS data, especially with four replicates or fewer (116).

The most informative experiment in assessing the impact of the various differences between our dataset and previously published datasets on protein complex identification has been provided by a SEC-MS study where the authors re-ran their samples using DDA instead of DIA (28). Despite separating the lysate into three times as many SEC fractions as our study (81 vs. 24), and using peptide-level rather than protein-level intensities as the data input, *CCprofiler* identified 298 CORUM protein complexes from their DDA-SEC-MS dataset—only modestly more than our 245 protein complexes and less than half of those observed when they had analyzed the same samples using DIA-SEC-MS (supplemental Fig. S3*D*). Therefore, DIA appears to be the major contributor to the higher protein complex identifications in the more recent published SEC-MS studies. If DDA must be used, protein- or peptide-level labeling workflows (*e.g.*, SILAC, tandem mass tag) could help to improve protein complex identification by reducing technical variability and/or missing values between conditions (118, 119, 120).

With more studies employing SEC-MS and other cofractionation-based MS approaches, we expect our dataset (*e.g.*, supplemental Table S2) to continue being of use as newer analysis pipelines, software, and best-practices are developed (121, 122, 123). Another useful future comparison would be SEC-MS with other clinically pursued HSP90 inhibitors—especially those that bind HSP90 outside of its N-terminal ATPase pocket, such as C-terminal domain binders of the novobiocin family (124) or covalent inhibitors targeting cysteine residues (125, 126). In a similar vein, inhibitors that target only a subset of the pan-HSP90 proteome by interfering with specific chaperone:co-chaperone interactions (*e.g.*, HSP90:CDC37 to target only protein kinase clients) (123), or only certain HSP90 isoforms (127, 128, 129, 130), are also being explored for clinical use. The ability of SEC-MS to identify remodeling events downstream of HSP90 in a global and unbiased manner should make this an attractive approach for rational development and deployment of next-generation HSP90 family inhibitors to the patients and pathological indications most likely to benefit.

## Data Availability

The raw MS proteomics data have been deposited to the ProteomeXchange Consortium (http://proteomecentral.proteomexchange.org/) *via* the PRIDE partner repository (35) with the dataset identifier PXD033459. This article contains Supplemental Data, with nine Supplemental Figures (Figs. S1–S9) and 10 Supplemental Tables (Tables S1–S10, uploaded as separate txt files).

## Supplemental data

This article contains supplemental data.

## Conflict of interest

The Institute of Cancer Research has a commercial interest in HSP90 and HSF1 inhibitors and operates a reward to discoverers scheme from which employees may benefit. P. W. received funding from Vernalis for the discovery of HSP90 inhibitors, and intellectual property for this program was licensed to Vernalis Ltd and Novartis. P. W. was previously involved in a research collaboration with AstraZeneca in the area of the HSF1 pathway, and intellectual property was licensed to Sixth Element Capital/Pioneer Fund and Nuvectis Pharma. P. W. has been/is a consultant/advisory board member to Alterome Therapeutics, Astex Therapeutics, Black Diamond Therapeutics, CHARM Therapeutics, CV6 Therapeutics, EpiCombi Therapeutics, Novartis, STORM Therapeutics, and Vividion Therapeutics (acquired by Bayer AG) and is a Science Partner for Nextech Invest. P. W. is a Non-Executive Board member and holds stock in STORM Therapeutics and also holds stock in Alterome Therapeutics, Black Diamond Therapeutics, Chroma Therapeutics, EpiCombi Therapeutics, Nextech Invest, and Nuvectis Pharma. P. W. is the Executive Director of the non-profit Chemical Probes Portal. P. W. and P. A. C. received research funding from Merck KGaA and Astex Therapeutics, and P. W. received research funding from AstraZeneca, Battle Against Cancer Investment Trust (BACIT), and CRT Pioneer Fund/Sixth Element Capital). P. W. is a former employee of AstraZeneca. B. A. L. is a former employee of The Institute of Cancer Research which operates reward to inventors program and a former employee of Inpharmatica Ltd (later acquired by Galapagos). B. A. L. has financial interest and/or acts/acted as a consultant or a Scientific Advisory Board member for Exscientia AI, AstraZeneca, Astex Pharmaceuticals, GSK, Astellas Pharma, and Definiens AG (member of AstraZeneca group). B. A. L. is the Director of Informatics for the non-profit Chemical Probes Portal.

## Supplemental Data

TableS1

TableS2

TableS3

TableS4

TableS5

TableS6

TableS7

TableS8

TableS9

TableS10
