# Supplementary material for: Native Size-Exclusion Chromatography–Based Mass Spectrometry Reveals New Components of the Early Heat Shock Protein 90 Inhibition Response Among Limited Global Changes
Source: Mol Cell Proteomics. 2022 Dec 20;22(2):100485. doi: 10.1016/j.mcpro.2022.100485 (PMC9898794; doi:10.1016/j.mcpro.2022.100485)
Supplement: Supplemental data [file mmc11.pdf]

Supplemental Data

Native Size-Exclusion Chromatography–Based Mass Spectrometry Reveals New Components of the Early Heat Shock Protein 90 Inhibition Response Among Limited Global Changes

doi: https://doi.org/10.1016/j.mcpro.2022.100485

R.S. Samant*‡, S. Batista‡, M. Larance, B. Ozer, C.I. Milton, I. Bludau, E. Wu, L. Biggins, S. Andrews, A. Hervieu, H.E. Johnston, B. Al-Lazikhani, A.I. Lamond, P.A. Clarke, P. Workman*

* For correspondence: Rahul S. Samant, rahul.samant@babraham.ac.uk; Paul Workman, paul.workman@icr.ac.uk.

‡ These authors contributed equally to this work.

LIST OF SUPPLEMENTAL MATERIAL IN THIS FILE

Supplemental Table Legends (page S-2)

Fig S1. Reproducibility between replicates (EXP1–EXP4) and differential protein analysis of summed intensities in SEC-MS dataset. (page S-3)

Fig S2. SEC-MS profiles of components of the Heat Shock Protein machinery. (page S-4)

Fig S3. Tight clustering of proteins within, but not between, regulatory particle (RP, 19S) and core particle (CP, 20S) of the 26S proteasome. (page S-5)

Fig S4. SEC profiles of all HSP90i-modulated protein complexes identified by PCprophet. (page S-6)

Fig S5. Protein:protein interactions networks identified in complete dataset. (page S-7)

Fig S6. Stringent DP protein:protein interaction network generated using canSAR curated interactome database. (page S-8)

Fig S7. Knockdown of Anillin by siRNAs reduces cell confluency and viable cell number. (page S-9)

Fig S8. Characterization of mitochondrial matrix protein cluster in Stringent DPs. (page S-10)

Fig S9. Uncropped images of immunoblots displayed in this manuscript. (page S-11)

LIST OF SUPPLEMENTAL TABLES (SEPARATE .txt FILES)

Table S1. Original proteinGroups.txt file from MaxQuant.

Table S2. Data-frame of 6,427 unique proteins.

Table S3. Data-frame of scaled intensities for the 6,427 unique proteins.

Table S4. Filtered data-frame of 4,645 proteins found in at least 3 of 4 experiments for either treatment condition.

Table S5. Differential expression analysis for summed intensities from 4,645 filtered proteins.

Table S6. Characterization of the 113 significant proteins (including 76 Differential Proteins, DPs) based on summed intensities, with respect to previous HSP90 inhibitor proteomics studies.

Table S7. Differential proteins identified by PCprophet.

Table S8. HSP90i-modulated protein complexes identified by PCprophet.

Table S9. Log_2-transformed Fold Change (LFC) values calculated by *R* package DEP for 366 All Fraction Differential Proteins (DPs).

Table S10. Log_2-transformed Fold Change (LFC) values calculated by *R* package DEP for 62 Stringent Fraction Differential Proteins (DPs).

SUPPLEMENTAL TABLE LEGENDS

Table S1. Original proteinGroups.txt file from MaxQuant. Data-frame of 7401 rows and 1595 columns. See MaxQuant documentation (http://www.coxdocs.org/doku.php?id=maxquant:table:proteingrouptable) for column descriptions.

Table S2. Data-frame of 6,427 unique proteins. Data-frame of 6,427 rows and 194 columns, containing HUGO classification Gene Names, Entrez Protein IDs, and all Label-Free Quantitation (LFQ) intensity values.

Table S3. Data-frame of scaled intensities for the 6,427 unique proteins. Data-frame of 6,427 rows and 194 columns, containing HUGO classification Gene Names, Entrez Protein IDs, and scaled intensity values. LFQ intensities from Table S2 were grouped by protein and replicate, and scaled between 0 and 1, i.e., '1' represents the fraction with the maximum intensity observed for that protein in that experiment (EXP1–EXP4), regardless of whether that maximum intensity fraction was in the Ctrl or HSP90i condition.

Table S4. Filtered data-frame of 4,645 proteins found in at least 3 of 4 experiments for either treatment condition. Data-frame of 4,645 rows and 194 columns, containing HUGO classification Gene Names, Entrez Protein IDs, and all LFQ intensity values.

Table S5. Differential expression analysis for summed intensities from 4,645 filtered proteins. Data-frame of 4,645 rows and 19 columns, containing: HUGO classification Gene Names ('Name'); Entrez Protein IDs ('ID'); $\log_2$-transformed LFQ intensities for each of the eight experiments; molecular mass in kDa ('mw_kDa'); whether or not any intensities were imputed for the analysis ('Imputed'); the number of missing values that needed to be imputed ('num_NAs'); confidence intervals (CI.L and CI.R); $\log_2$-transformed fold change ('LFC'); Benjamini-Hochberg-corrected adjusted *p*-values ('p.adj'); unadjusted Student's *t*-test *p*-values ('p.val'); whether or not the protein is identified as significant based on p.adj < 0.05 ('Significant'); and whether or not the protein is identified as a Differential Protein, based on p.adj < 0.05 and LFC > 1 or < -1 ('DP').

Table S6. Characterization of the 113 significant proteins (including 76 Differential Proteins, DPs) based on summed intensities, with respect to previous HSP90 inhibitor proteomics studies. Data-frame of 113 rows by 18 columns, containing: HUGO classification Gene Names ('Name'); Entrez Protein IDs ('ID'); alternative names for protein ('Alt_names'); $\log_2$-transformed fold change from DEP analysis ('LFC'); Benjamini-Hochberg-corrected adjusted *p*-values from DEP analysis ('p.adj'); unadjusted Student's *t*-test *p*-values from DEP analysis ('p.val'); whether or not the protein is identified as significant based on p.adj < 0.05 ('Significant'); whether or not the protein is identified as a Differential Protein, based on p.adj < 0.05 and LFC > 1 or < -1 ('DP'); whether the protein increased or decreased in abundance upon HSP90 inhibition ('Direction'); whether the protein was identified as a DP in previous HSP90 proteomics studies ('Previously_Identified_DP'), whether the protein was identified as a DP (TRUE), not identified as a DP (FALSE), or not identified at all (NA) in the specific study ('Savitski2018', 'Quadroni2015', 'Fierro-Monti2013', 'Voruganti2013', 'Sharma2012', 'Wu2012'); whether or not the protein was identified experimentally as an interactor of human HSP90AA1 or HSP90AB1 in the Hsp90 PPI database (https://www.picard.ch/Hsp90Int/)('PicardList'); whether or not the protein was identified as an HSF1 target gene (https://hsf1base.org/)('HSF1_target').

Table S7. Differential proteins identified by PCprophet. Data frame of 5,199 by 4 columns, containing: HUGO classification Gene Names ('Name'); PCprophet-calculated 'Abundance_log_marginal_likelihood' for null and alternative hypotheses; and 'Probability_differential_abundance', with values > (but not equal to) 0.5 representing differential proteins.

Table S8. HSP90i-modulated protein complexes identified by PCprophet. Data frame of 320 by 24, containing: CORUM 'ComplexID'; protein subunits from ComplexID identified as co-eluting ('Members'); PCprophet-calculated 'Abundance_log_marginal_likelihood' for null and alternative hypotheses; 'Probability_differential_abundance', with values > (but not equal to) 0.5 representing differential complexes; CORUM 'ComplexName'; 'Organism' complex was identified in; alternative names for complex ('Synonyms'); 'Cell line' complex was identified in; subunits(UniProt IDs); subunits(Entrez IDs); protein complex purification method; GO description; FunCatID; FunCat description; subunits(Gene name synonyms); Complex comment; Disease comment; SWISSPROT organism; Subunits comment; gene names of all subunits annotated in the complex ('subunits(Gene name)'); PubMed ID; full protein name of all subunits in the complex ('subunits(Protein name)').

Table S9. $\log_2$-transformed Fold Change (LFC) values calculated by *R* package DEP for 366 All Fraction Differential Proteins (DPs). Data-frame of 366 rows by 25 columns, containing: HUGO classification Gene Names ('Name') and $\log_2$-transformed fold change from DEP analysis in each fraction ('LFC_F01'–'LFC_F24').

Table S10. $\log_2$-transformed Fold Change (LFC) values calculated by *R* package DEP for 62 Stringent Fraction Differential Proteins (DPs). Data-frame of 62 rows by 28 columns, containing: HUGO classification Gene Names ('Name'); Markov cluster ID ('Cluster_Number') and colour ('Cluster_Colour') from STRING network, as illustrated in Fig 4C; number of nodes present in the Markov cluster ('Node_Count'); and log2-transformed fold change from DEP analysis in each fraction ('LFC_F01'–'LFC_F24').

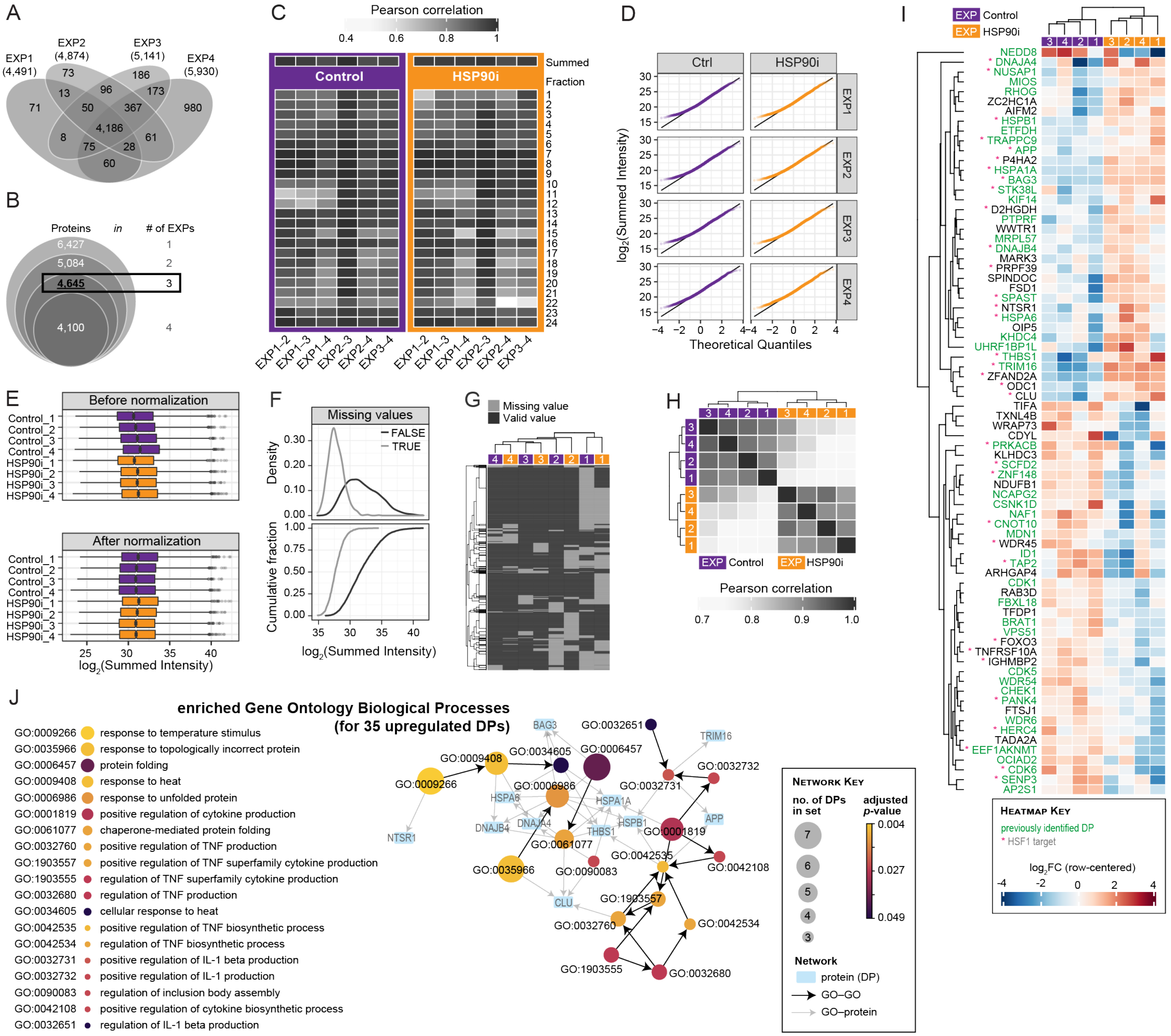

Figure S1. Reproducibility between replicates (EXP1–EXP4) and differential protein analysis of summed intensities in SEC-MS dataset. (**A**) Venn diagram showing overlap between the proteins identified in each biological replicate (EXP1–EXP4). (**B**) Venn diagram to show total number of proteins identified in 1, 2, 3, or 4 biological replicates. For (A) and (B), proteins identified in either Control or HSP90i conditions were combined. (**C**) Heatmap showing Pearson correlation coefficients between the four biological replicates for each of the 24 SEC fractions, individually and summed, under both experimental conditions. (**D**) Quantile-quantile plots of log_2-transformed summed LFQ intensities from filtered dataset (4,645 proteins) indicate a similar heavy-tailed distribution in all experiments. This type of distribution is typical in mass-spectrometry-based proteomics data. (**E**) Box/Tukey plots showing spread of summed intensities for each experiments before and after background correction and variance-stabilizing transformation. (**F**) Linegraphs showing densities (top) and cumulative fractions (bottom) of log_2-transformed summed intensities for proteins with (TRUE) and without (FALSE) missing values. Data indicate that proteins with missing values tend to have lower summed intensities. (**G**) Heatmap of entries from 4,645 filtered proteins list with at least one missing value across the eight samples. (**H**) Heatmap summarizing Pearson correlation coefficients for summed intensities across all experiments. (**I**) Heatmap showing row-centered intensities for all 76 significant differential-expressed proteins based on summed intensity values. Clusters are drawn from Euclidean distance. Heatmap and plots in (D)–(G) were generated from built-in functions in *R* package 'DEP'. (**J**) Gene Ontology Biological Processes (GOBPs) significantly enriched among the 35 upregulated differential proteins (DPs) identified in Fig 1F using GOnet, with the 4,645 filtered proteins used as the background for the enrichment analysis. DPs linked to the enriched GO terms are shown.

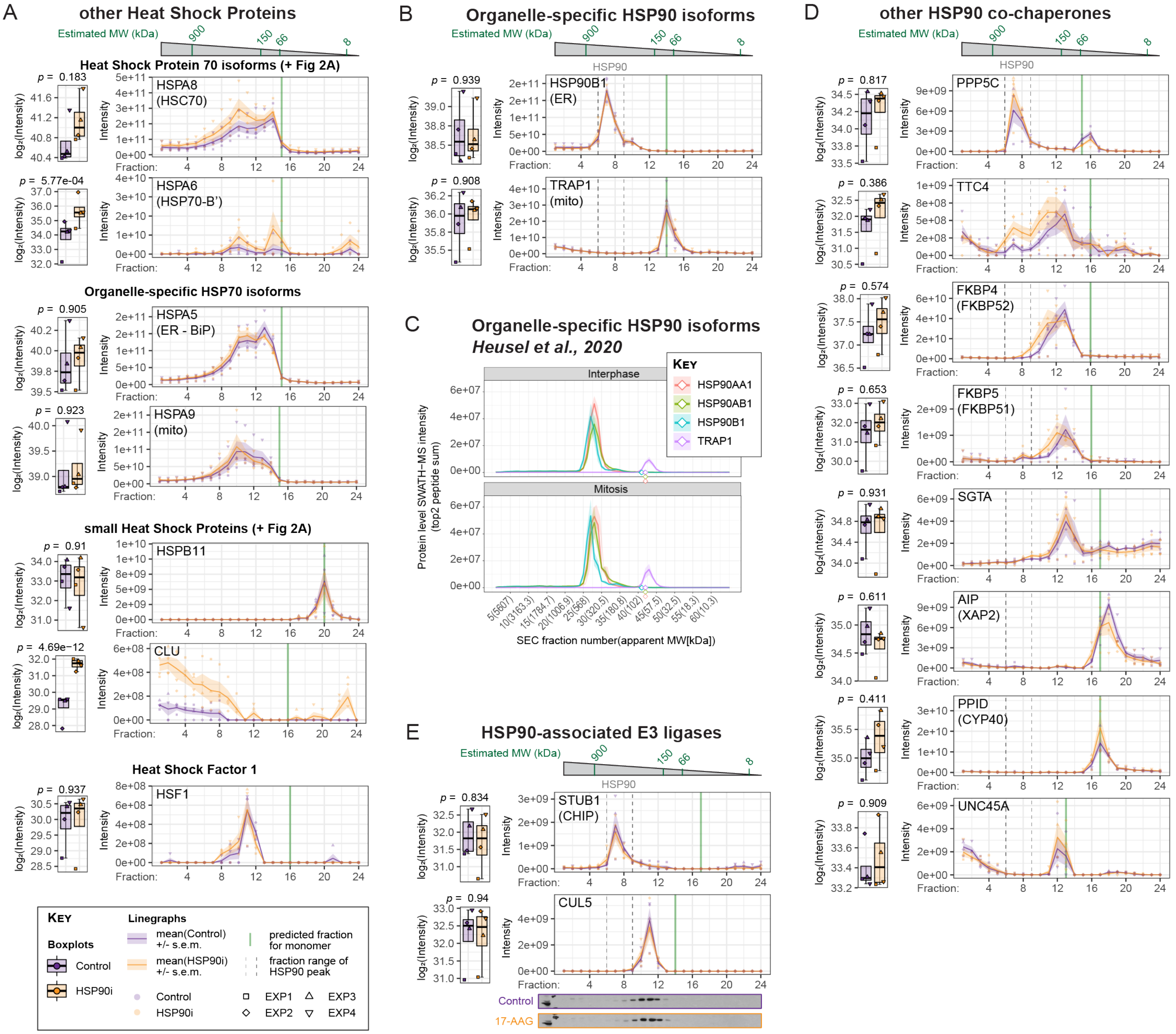

Figure S2. SEC-MS profiles of components of the Heat Shock Protein machinery. (**A**) SEC-MS profiles of other heat shock-induced proteins and their transcriptional activator HSF1. (**B**) SEC-MS profiles of organelle-specific HSP90 isoforms HSP90B1/GRP94 (endoplasmic reticulum) and TRAP1 (mitochondria). (**C**) SEC-SWATH-MS profiles from Heusel et al., 2020, for HSP90 isoforms HSP90AA1/HSP90α, HSP90AB1/HSP90β, HSP90B1/GRP94 (endoplasmic reticulum) and TRAP1 (mitochondria), from interphase and mitotic HeLa-CCL2 cells. Profiles were generated using SECexplorer-cc (https://sec-explorer.shinyapps.io/hela_cellcycle/). Solid lines represent mean values for the top two peptides across three replicated for each protein. Shaded areas represent the standard error of the mean. Diamonds indicate estimated fraction for the monomer peak of each protein, based on the UniProt-annotated molecular weight. (**D**) SEC-MS profiles of other tetratricopeptide (TPR)-domain co-chaperones of HSP90. Only PPP5C/PP5 had a major elution peak in the same fractions as HSP90. (**E**) SEC-MS profiles of two E3 ubiquitin ligases known to be recruited to HSP90 following HSP90 inhibition. Only STUB1/CHIP co-eluted with HSP90. Neither E3 ubiquitin ligase displayed an altered profile following HSP90 inhibition.

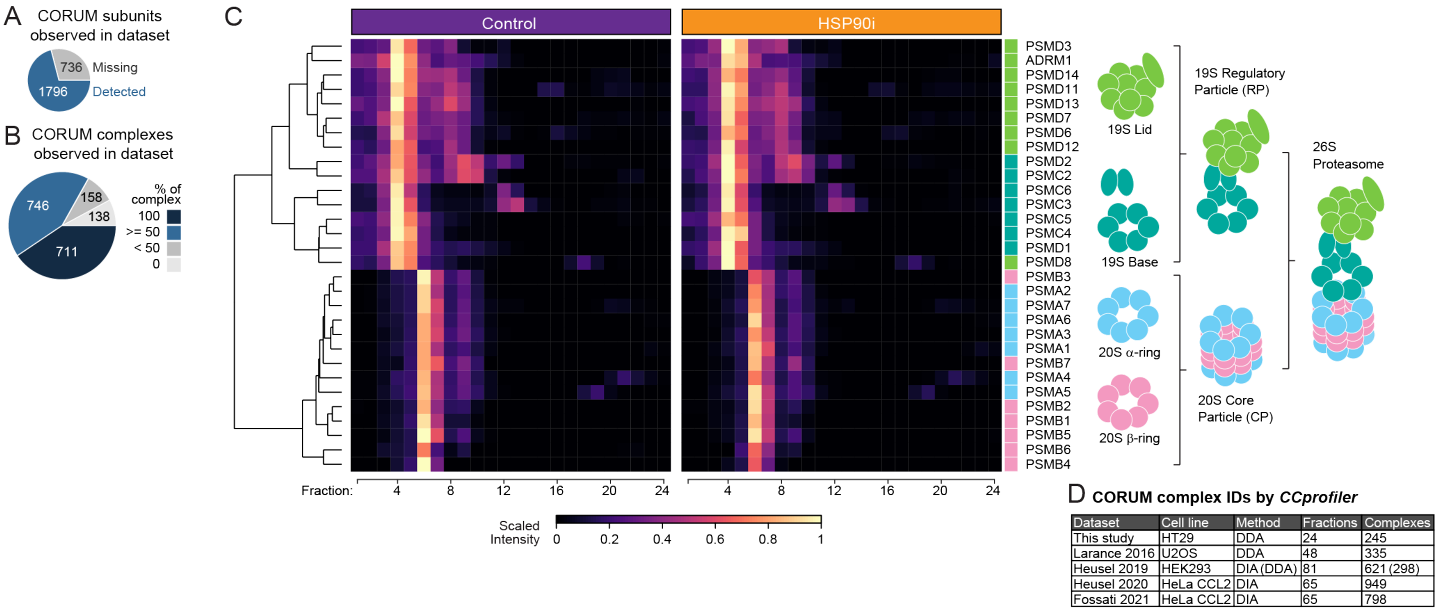

Dataset	Cell line	Method	Fractions	Complexes
This study	HT29	DDA	24	245
Larance 2016	U2OS	DDA	48	335
Heusel 2019	HEK293	DIA (DDA)	81	621 (298)
Heusel 2020	HeLa CCL2	DIA	65	949
Fossati 2021	HeLa CCL2	DIA	65	798

Figure S3. Tight clustering of proteins within, but not between, regulatory particle (RP, 19S) and core particle (CP, 20S) of the 26S proteasome. (**A–B**) Pie charts illustrating the number of proteins (A) and protein complexes (B) annotated in the CORUM protein complex database that were covered in our dataset of 6,427 proteins. (**C**) Heatmap of scaled intensities for subunits of the 19S regulatory particle and 20S core particle complexes that make up the 26S proteasome. The dendogram cut-offs based on Euclidean distance matrix with the Ward-D2 linkage method are illustrated to the left of the heat map. (**D**) Benchmarking the number of CORUM-annotated protein complexes identified by *CCprofiler* from our dataset compared with previous published SEC-MS studies. The cell line, MS acquisition method (data-dependent acquisition, DDA, or data-independent acquisition, DIA), number of SEC fractions collected, and number of protein complexes identified by *CCprofiler* (using a cut-off of >= 50 % of subunits co-eluting) are indicated. The number of complexes identified are according to those reported in each study, except for Larance et al., which pre-dates *CCprofiler*, and was re-analyzed using *CCprofiler* in Heusel et al. 2019 (https://doi.org/10.15252/msb.20188438).

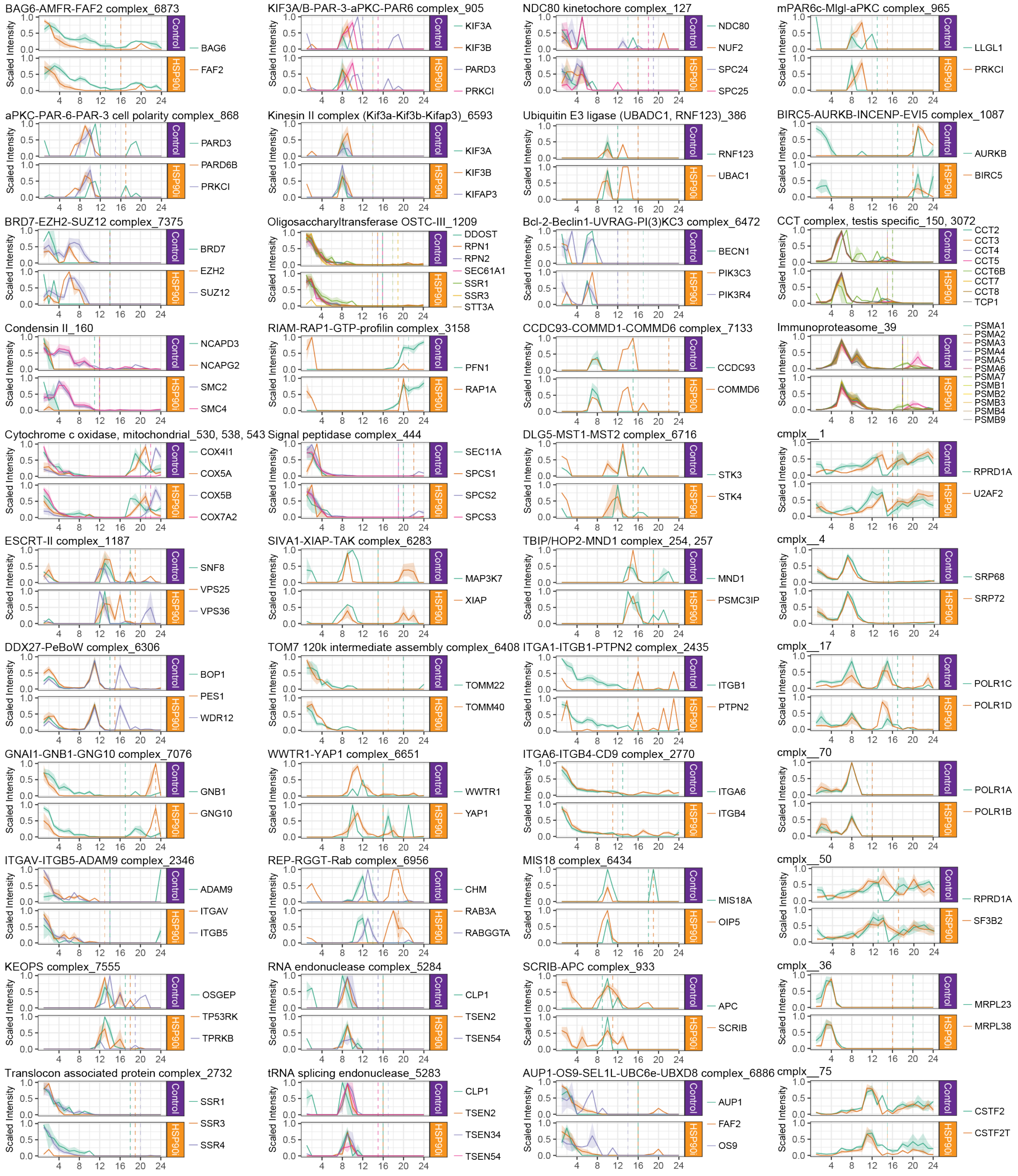

Figure S4. SEC profiles of all HSP90i-modulated protein complexes identified by PCprophet. Scaled median intensities for all subunits from the protein complex identified as co-eluting by PCprophet were plotted. CORUM ID numbers are indicated in the title. For complexes with multiple CORUM IDs, only one graph was plotted (e.g., 'Cytochrome c oxidase, mitochondrial' CORUM IDs 530, 538, and 542; 'CCT complex, testis specific' CORUM IDs 150 and 3072). Novel protein complexes not annotated in CORUM are indicated with the prefix "cmplx". Dashed vertical lines on linegraphs indicate the fraction in which the monomer would be detected, based on the UniProt-annotated molecular weight.

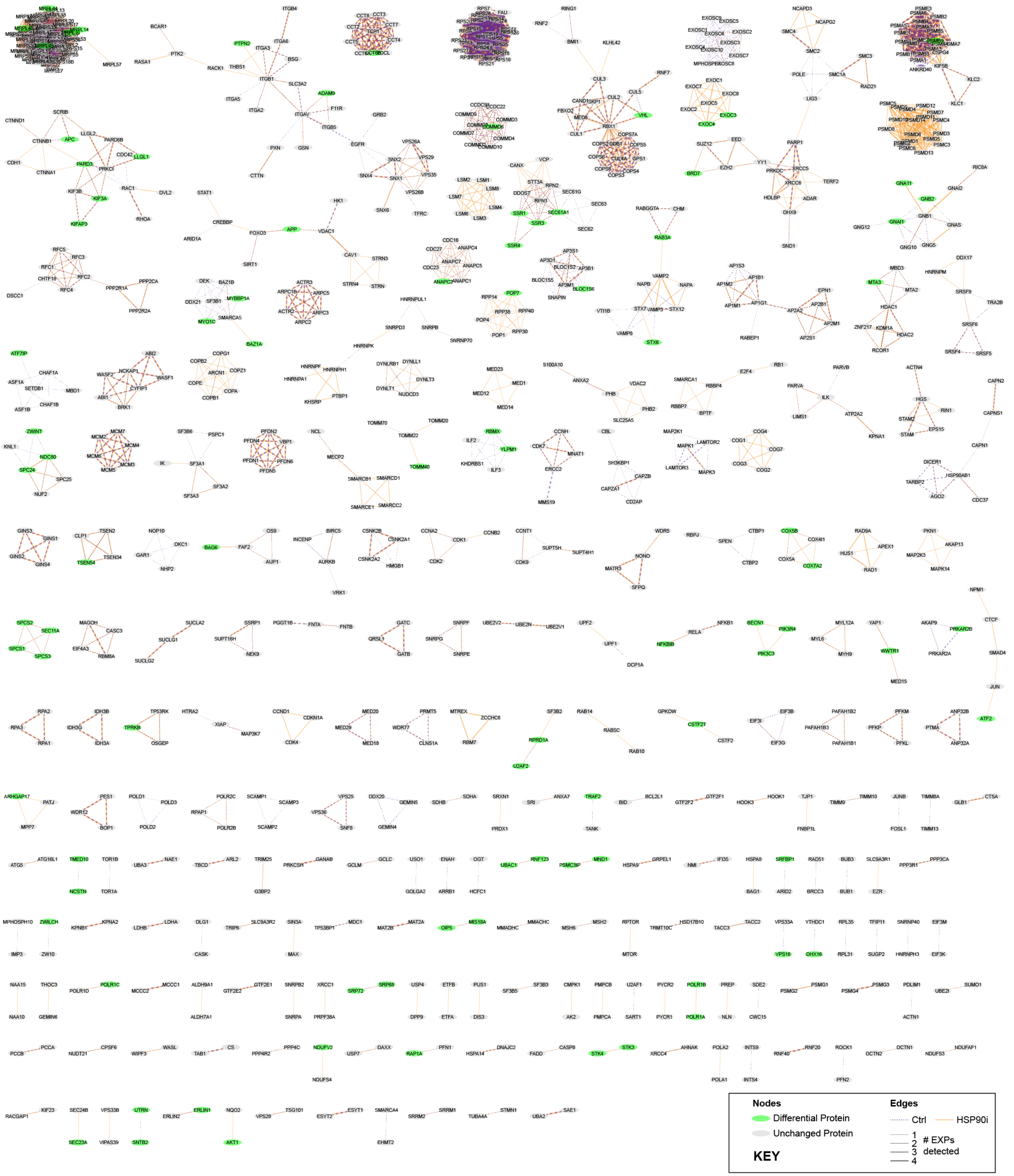

Figure S5. Protein:protein interaction networks identified in complete dataset. Protein:protein interaction networks generated by PCprophet based on our complete dataset of 6,427 proteins. Networks were generated using Cytoscape based on ‘PPIreport.txt’ PCprophet output. Green nodes represent differential proteins identified using PCprophet’s protein-centric analysis. Edge width represents the number of experiments in which the interaction was confidently detected by PCprophet, with the edge colour representing Ctrl or HSP90i detections.

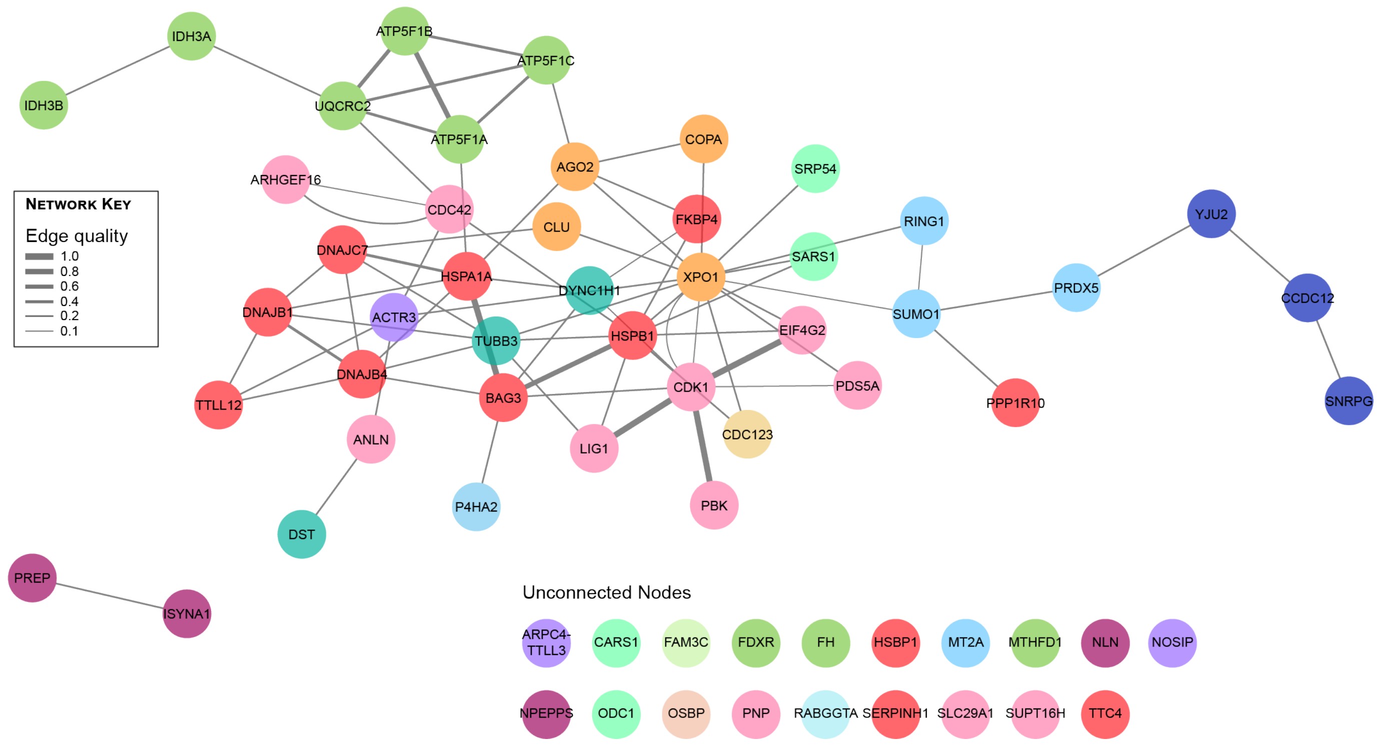

Figure S6. Stringent DP protein:protein interaction network generated using canSAR curated interactome database. The list of 62 stringent fraction DPs from Fig 4 were entered into the canSAR Protein Annotation Tool (https://cansarblack.icr.ac.uk/cpat), with edges representing interactions from canSAR's curated interactome. Edge width represents confidence of evidence ('edge quality') for interaction between the two nodes. Node colours represent clusters from Fig 4.

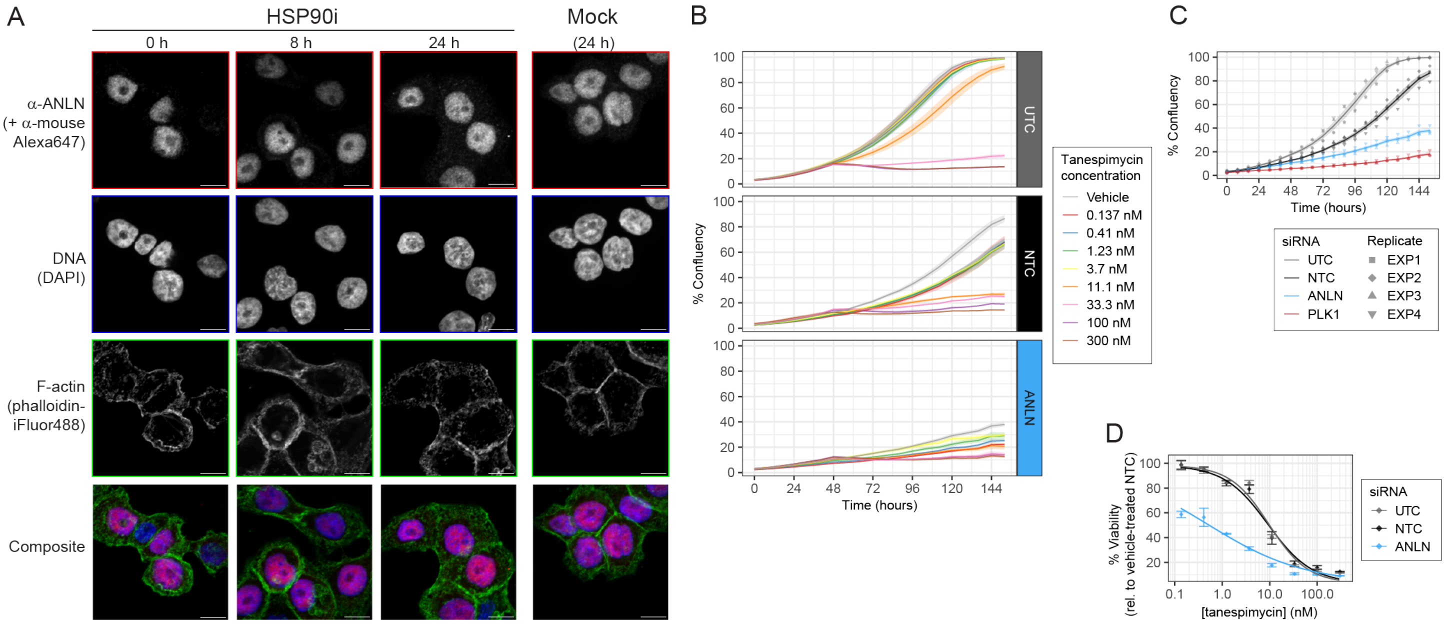

Figure S7. Knockdown of Anillin by siRNAs reduces cell confluency and viable cell number. (**A**) Anillin remains predominantly nuclear upon HSP90 inhibition. HT29 cells seeded on gelatin-coated glass coverslips overnight were treated with 62.5 nM tanespimycin, or mock treated with DMSO vehicle, for the time indicated before fixation with 4 % paraformaldehyde for 15 min at room temperature. Cells were permeabilized (0.1 % Triton X-100, 10 min) and stained for Anillin, F-actin, and DNA (all at room temperature), before mounting (ProLong^{TM} Glass Antifade Mountant) and imaging using a Nikon A1R confocal microscope. Images represent maximum intensity projections from z-stacks. Scale bars = 10 µm. (**B, C**) Knockdown of Anillin reduces HT29 cell confluency compared with non-targeting control (NTC) and untargeted control (UTC). HT29 cells were treated for 48 h with siTOOLs pool of 30 siRNAs (25 nM total concentration) targeted against Anillin (ANLN), non-targeting control NTC, untreated control UTC, or death-inducing control PLK1, followed by 96 h with a range of tanespimycin concentrations (except for PLK1), or with mock treated with vehicle control (0.1 % DMSO), while still in the presence of the original siRNAs. Confluency was monitored every 8 h throughout the time-course by Incucyte®. Lines and shaded regions represent mean +/- standard error from four biological replicates (EXP1–EXP4) for each condition. (**C**) Confluency measurements for vehicle control-treated cells from each siRNA condition in **A**. (**D**) Knockdown of Anillin reduces number of viable HT29 cells compared with non-targeting control (NTC) and untargeted control (UTC). Percentage viability of HT29 cells from part **B** at the end of the siRNA and tanespimycin treatments (144 h total) by CellTiter-Blue® assay relative to vehicle-treated (0.1 % DMSO) NTC control was measured with an Incucyte®. Points and error bars represent mean +/- standard error for each condition. Dose-response curve fitting was performed using the 'Log[Inhibitor] *vs.* normalized response – Variable slope' non-linear regression model in Graphpad Prism.

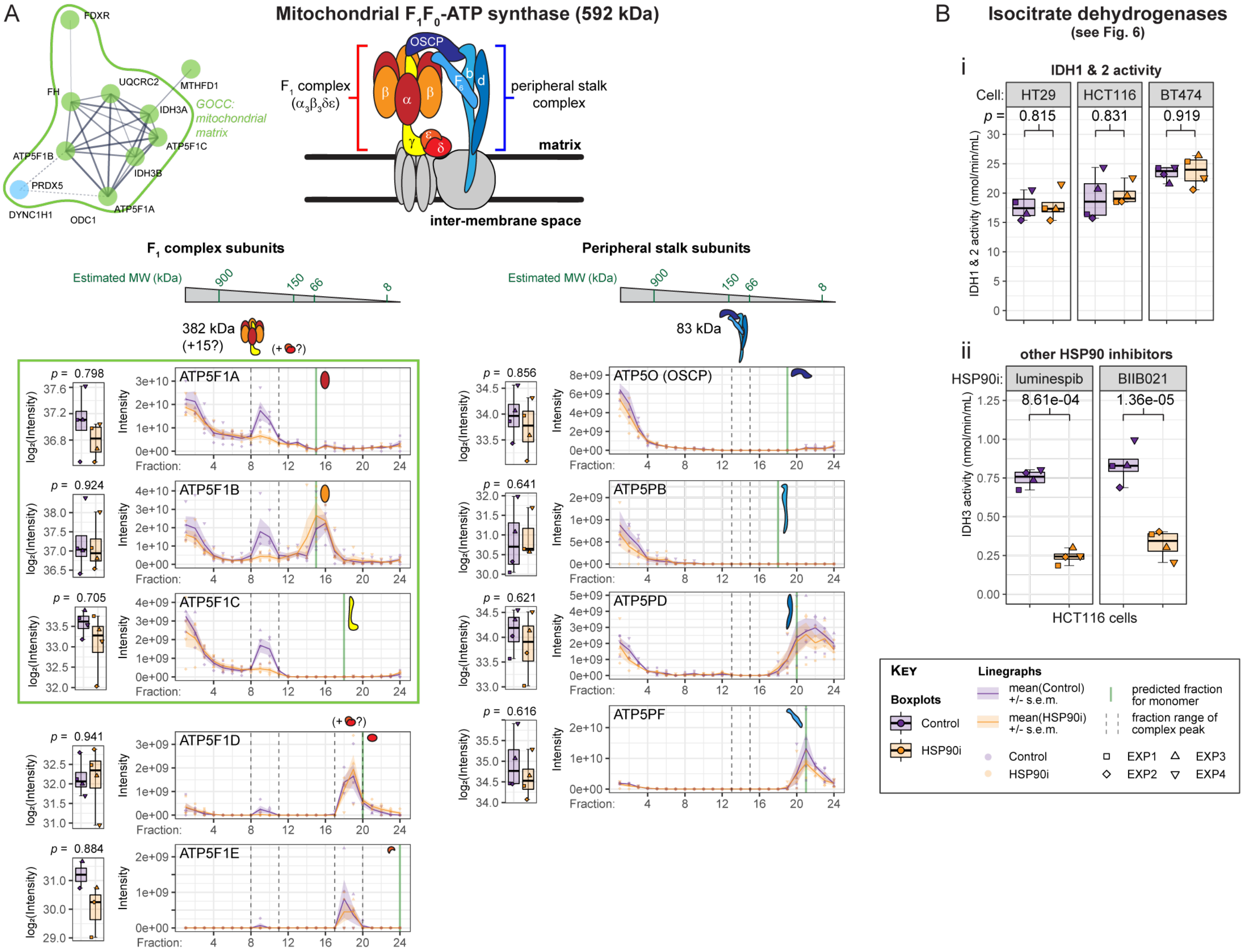

Figure S8. Characterization of mitochondrial matrix protein cluster in Stringent DPs. (**A**) Top: Focusing on the mitochondrial matrix protein cluster within the Stringent DP network from Fig 4B, we identify three of the five subunits comprising the F_1 sub-complex from the mitochondrial F_1F_0-ATP synthase complex. Bottom Left: SEC-MS profiles of the three enriched ATP synthase F_1 subunits, as well as the other two non-enriched subunits, showing clear co-elution at the predicted molecular weight of the ATP5F1A:ATP5F1B:ATP5F1C sub-complex in the Control but not the HSP90i condition. Note that it is unclear whether a sub-population of the non-enriched subunits ATP5F1D and/or ATP5F1E also co-elute in the Control condition. Bottom Right: SEC-MS profiles of the four peripheral stalk subunits show no co-elution at the predicted sub-complex molecular weights. (**B**) Activity of the mitochondrial Isocitrate Dehydrogenase 3 (IDH3) complex, but not IDH1 or IDH2, is reduced by treatment with multiple HSP90 inhibitors. (i) Combined activities of IDH1 & IDH2 are not significantly reduced upon HSP90 inhibition in HT29 colon adenocarcinoma, HCT116 colon carcinoma, and BT474 breast ductal carcinoma cell lines. Activity was measured using the IDH Activity Assay Kit (Sigma) according to manufacturer's instructions, using $NADP^+$ as the co-factor—which is used by both IDH1 and IDH2. See Fig 6B for corresponding assay with NAD^+ as the co-factor, for estimating IDH3 activity. (ii) IDH3 activity is significantly reduced upon treatment with the chemotypically distinct HSP90 inhibitors luminespib (AUY922) and BIIB021, in HCT116 colon carcinoma cells. Activity was measured using the IDH Activity Assay Kit (Sigma) according to manufacturer's instructions, using NAD^+ as the co-factor.

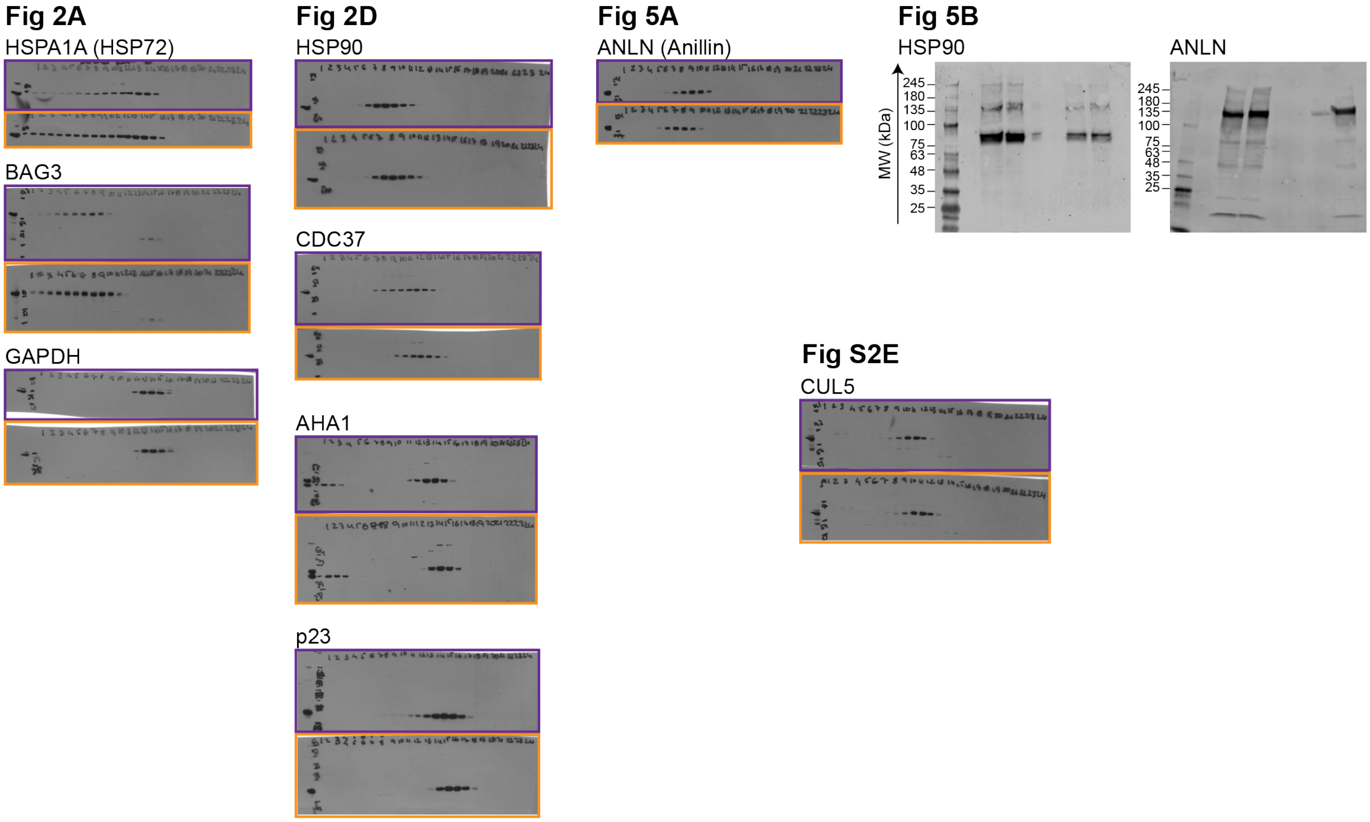

Figure S9. Uncropped images of immunoblots displayed in this manuscript. See 'SEC-Immunoblotting' section in Experimental Procedures of main article for antibodies and dilutions used.
